# Predicting transitions across macroscopic states for railway systems

**DOI:** 10.1371/journal.pone.0217710

**Published:** 2019-06-06

**Authors:** Mark M. Dekker, Debabrata Panja, Henk A. Dijkstra, Stefan C. Dekker

**Affiliations:** 1 Department of Information and Computing Sciences, Utrecht University, Princetonplein 5, 3584 CC Utrecht, The Netherlands; 2 Institute for Marine and Atmospheric research Utrecht, Department of Physics, Utrecht University, Princetonplein 5, 3584 CC Utrecht, The Netherlands; 3 Centre for Complex Systems Studies, Utrecht University, Minnaertgebouw, Leuvenlaan 4, 3584 CE Utrecht, The Netherlands; 4 Copernicus Institute of Sustainable development, Utrecht University, Princetonlaan 8a, 3584 CB Utrecht, The Netherlands; Universidad Rey Juan Carlos, SPAIN

## Abstract

Railways are classic instances of complex socio-technical systems, whose defining characteristic is that they exist and function by integrating (continuous-time) interactions among technical components and human elements. Typically, unlike physical systems, there are no governing laws for describing their dynamics. Based purely on micro-unit data, here we present a data-driven framework to analyze macro-dynamics in such systems, leading us to the identification of specific states and prediction of transitions across them. It consists of three steps, which we elucidate using data from the Dutch railways. First, we form a dimensionally reduced phase-space by extracting a few relevant components, wherein relevance is proxied by dominance in terms of explained variance, as well as by persistence in time. Secondly, we apply a clustering algorithm to the reduced phase-space, resulting in the revelation of states of the system. Specifically, we identify ‘rest’ and ‘disrupted’ states, for which the system operations deviates respectively little and strongly from the planned timetable. Third, we define an early-warning metric based on the probability of transitions across states, predict whether the system is likely to transit from one state to another within a given time-frame and evaluate the performance of this metric using the Peirce skill score. Interestingly, using case studies, we demonstrate that the framework is able to predict large-scale disruptions up to 90 minutes beforehand with significant skill, demonstrating, for the railway companies, its potential to better track the evolution of large-scale disruptions in their networks. We discuss that the applicability of the three-step framework stretches to other systems as well—i.e., not only socio-technical ones—wherein real-time monitoring can help to prevent macro-scale state transitions, albeit the methods chosen to execute each step may depend on specific system-details.

## 1 Introduction

Railways are classic examples of complex socio-technical (ST) systems. Their defining characteristic is that they integrate (continuous-time) interactions among technical components and human elements/influence in their existence and functionality. In this paper, we think of ST systems as dynamical systems, although, typically there are no laws that govern their time evolution. There exists a substantial amount of literature to model the behavior of ST systems; e.g., on innovation [1, 2], the performance of medical services [3], spread of diseases [4], agri-food systems [5] and infrastructure [6] or social media networks [7]. Ranging from agent-based models [5] to more analytical network-diffusion ones [8], various model frameworks have been proposed for describing their time evolution. However, to the best of our knowledge, less attention has been paid to developing data-driven frameworks to analyze their dynamical properties.

One common denominating factor for ST systems is the ubiquity of the accumulated heterogeneous spatio-temporal data: numerous points in (network-)space own a time series of measurements, constituting a ‘signal’. For railways, the subject of this paper, this signal is the accumulated delay of trains that (should) pass by a part of the network. In epidemiology, the signal is the number of infections in a city or a region. In social networks, the signal can refer to, for instance, a video, an opinion or a Twitter hash tag. The aspects of heterogeneity, absence of physical laws and the ubiquity of spatio-temporal data can also be attributed to many other (non-ST) systems, notably in neuroscience. The natural questions that are central to all of these systems are: how do the signals evolve (e.g., development of a communicable disease to a pandemic, or large-scale disruptions in railway systems)? In particular, can one define specific macro-states for these systems, and describe transitions across such states? In this paper we focus on these questions.

In physical systems, transitions are widely discussed topics. Therein, transitions are often associated with bifurcation points, inducing multiple equilibria or limit cycles [9–11]. For the analysis of such bifurcation points, the underlying equations should (at least partly) be known, which is generally not the case for ST systems. In contrast, working with observations of such a system usually involves dealing with noise and filtering long-range correlations from time series that at first sight do not show any trend or approximation of a transition regime. The behavior of such a system close to (standard) transitions is referred to as ‘critical slowing-down’, and can often be traced to an increased variance and autocorrelation in time [12, 13]. More advanced techniques, especially when dealing with different time scales in the data, are degenerate fingerprinting and detrended fluctuation analysis [14, 15]. Applications of these techniques can be found in, for example, vegetation systems [16] and climate variability [17].

In physical systems too, the underlying dynamics or equilibrium structure may not always be known; modeling of such systems calls for the development of data-driven frameworks. An example of such a data-driven analysis of a physical system is [18], where two different regimes in atmospheric northern hemisphere flow were analyzed. Using a reduced phase-space obtained through principal component analysis, the dynamics of the system was therein analyzed using the properties of transition matrices, which are determined using Ulam’s method [19]. Transition matrices as part of forward integration simulation models have also been used by [20], approximating ocean surface circulation using buoy data to simulate the movement of marine plastics in time. In this paper we generalize and combine some of these data-driven approaches for ST systems.

Our framework is showcased by data made available by the Dutch railways. Physical properties of a railway system are, for example, the velocity of trains, (dynamic) network capacity and the locations of switches and connections. Human elements in the system range from local dispatching and driving of trains and passengers to macro-scale decision making on the cancellation or rerouting of trains. Like in many ST systems, the interplay between the two causes prediction difficulties, with questions like: ‘In what circumstances will people cancel a train?’ or ‘How does delay of a certain train affect others if it gets rerouted?’.

For railways, it is highly relevant to be able to understand and predict the propagation of delay. If prediction were to be accurate, many disruption management techniques have been proposed in literature, involving research on the timetable adjustment [21], and rolling stock and crew rescheduling [22, 23].

**Fig 1:**
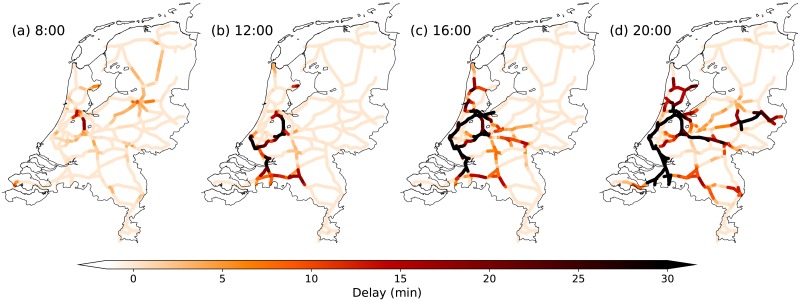
Dutch railway network in colored lines (thin black lines depict the coastal and country borders of the Netherlands). Coloring indicates delay per segment on the Dutch railway network, at four instances on February 3rd 2012. Figure similar as in Ref. [24], which discusses this day in more detail.

While any day contains numerous small fluctuations, specific combinations of delayed trains and external factors can build up to severe, nation-wide disrupted situations, as described for several instances of the Dutch railways in [24]. The winter of 2012 contained several of these nation-wide disrupted days for the Dutch railways. One of them is shown in Fig 1, where a clear propagating signal of high accumulated-delay is visible. An investigation conducted by the Dutch ministry of infrastructure concluded that a series of unfortunate coincidences were the cause for the disruption on that day, with 2-3 times higher-than-usual infrastructure disruptions and delay caused by missing personnel [25]. This is a classic example of the emergence of a (disrupted) macro-state, built up by micro-interactions (e.g., one train affecting the next, and so on). To the knowledge of the authors, there is no evidence for critical slowing down prior large disruptions in the railway systems, probably due to the heterogeneous and discrete nature of the variables.

Although many disturbances (caused by, e.g., accidents or ill personnel) are by definition unpredictable, the subsequent evolution of a primary delay source may be. Approaches in transportation science to investigate this can roughly be split into (a) modeling studies (for the testing of time-tables, and first-order robustness of the railway system, modeling studies are vital), and (b) data-driven studies on micro-interactions. Modeling of delay propagation is done at various levels, although they typically leave out human influence on the system, like decision making by the dispatchers. Micro-simulation modeling tools are often used to test time-tables, and simulate first-order delay propagation, involving tools like FRISO [26] and OpenTrack [27]. More abstract modeling studies involve analytical approaches to system equilibria [28] and first-order delay propagation [29, 30]. Agent-based models, too, are used to simulate railway systems [31], or gaming studies to back up the behavior of agents [32].

The key advantage of data-driven studies, in contrast to the above, is that all interactions and human influence are fully captured by the data. The main disadvantages is, of course, that it is difficult—if not impossible—to disentangle physical processes from human or case-dependent influence, specifically those processes that are robust (in many cases the same), rather than incidental (unique per case). Examples of data-driven studies in railways literature have mostly been performed at the micro-scale, e.g., the statistical estimation of specific train activities like running and dwell times [33–36]. Machine learning techniques like support vector machines are use to predict train arrival times from data in Serbia [37] and Italy [38]. Instances of combining data-driven and modeling approaches for railways systems exist too, helping to extract processes (like physical ‘laws’) from data [6], which has led to the identification of delay propagation patterns for the Italian and German railways, and eventually to the modeling of the dynamics like backward propagation of delays. A recent study to predict delay propagation uses hybrid Bayesian network models, focusing on one high-speed train line in China [39], while another combines Bayesian networks with stochastic prediction by updating the probability distribution from which future train delays are drawn [40].

What is currently still missing in railways literature is a data-driven framework to analyze and predict evolutions in delay at the meso- (regional) as well as the macro- (entire system) scale (as in Fig 1). With reference to the focus of this paper, we note that most data-driven studies, being mainly focused on the micro-scale, fail to reproduce (or are made for another scope) emergent phenomena like transitions across macro-states. Given that the means to predict these major events is of high societal relevance, the framework we present in this paper demonstrates the potential to successfully address this issue.

Our framework consists of three steps and is described in Sec. 2. We start with dimension reduction by applying principal component analysis to the data, where a small set of components is chosen based on variance and persistence in time to define a reduced phase-space. This is followed by clustering on this phase-space, resulting in a (near-automatic) identification of the ‘rest’ and ‘disrupted’ states. The third step consists of applying an early warning procedure that allows for real-time forecasting of the system, specified to predict the evolution towards the disrupted state. In the subsequent sections, we couple the framework to data made accessible by the Dutch railways: we introduce the data and the general results of the framework in Sec. 3, and present the results of two case studies in Sec. 4. We end the paper with a summary and concluding remarks in Sec. 5.

## 2 The three-step framework

As mentioned above, many ST systems concern spatio-temporal data: numerous points in (network-)space that own a time series, constituting a ‘signal’. For the railways (as we will discuss in section 3), this signal is the accumulated delay of trains that (should) pass by a part of the network.

### 2.1 Step 1—Dimension reduction

Given the spatio-temporal data, the first step concerns capturing the dynamics of the system in a few relevant system descriptors that define a reduced phase-space (while simultaneously saving computational efforts and time). Given the spatial dimension *N* (i.e., the number of nodes/grid-points where system variables are measured), and temporal dimension *M*, the matrix *D* containing all data for the system has dimension *N* × *M*. For many dimension reduction schemes, the averages of each time series is subtracted before *D* is computed.

An example of such a dimension reduction scheme is principal component analysis (PCA), which is the method we use for the railways. PCA finds orthogonal vectors, expressed as linear sums of the *N* time series, optimized for the portion of variance they explain. In mathematical terms, when the full time-series is involved, this entails the diagonalization of the correlation matrix *D*^*T*^ *D* (*N* × *N* matrix, superscript *T* denoting the transpose): i.e., expressing *D*^*T*^ *D* = *U*Σ*V*^*T*^ (note that *U* = *V*, since *D*^*T*^ *D* is real and symmetric). The columns of *V* are the eigenvectors of *D*^*T*^ *D*, commonly referred to as the principal components (PCs). The elements of the columns of *V* represent co-varying parts of the system (in space) that are known as empirical orthogonal functions (EOFs), while Σ is a diagonal matrix containing the eigenvalues of *D*^*T*^ *D* or the variances of the PCs. By construction, every snapshot of the full system, i.e., the system-wide values of the time-series data at any instant of time, can be expressed as a sum of the PCs with amplitudes. In other words, the PCs define the phase-space for the system, and the dynamics of the system is then described by the time evolution of the amplitude of the PCs.

One important disadvantage of PCA is that it is built to retrieve patterns that optimize the explained variance, while in practice these patterns might not be the most relevant ones. For some systems, such as in neuroscience, high-variance patterns may simply concern uninteresting features. Further, the most interesting signals may not be orthogonal to each other, which PCA enforces. For such systems generalized eigenvalue analysis may be more suitable [41].

PCA does work for the railway system, but it is important to find those PCs that are the most suitable to capture the development of large-scale disruptions. For this reason, first of all, PCs gained from the full dataset *D* are not the best ones. Instead, (as we will see in Sec. 3) a subset of full the time series that hold the data on ‘disrupted days’ is more suitable, resulting in a matrix *D*′ with dimensions *N* × *M*′, with *M*′ < *M*. Secondly, in order to describe the system’s dynamics in a reduced phase-space, the right PCs need to be retained (and the rest discarded). We find that in choosing the right PCs for the railways, we need to consider (a) the amount of variance explained by these PCs, and (b) the persistence of their amplitudes in time. We will take these up in Sec. 3.

### 2.2 Step 2—Identification of macro-states

The goal of the second step is to identify macro-states within the reduced phase-space. The macro-states are system-dependent. In case of infectious disease spreading, they may include a state with no infectious and a state involving a large-scale epidemic or pandemic. For railways, we can distinguish two types of states: ‘rest’ states, where the system largely adheres to the timetable, and ‘disrupted’ states. Depending on where in the network the delay is concentrated, the latter can take many different forms.

These macro-states can in general be approximated by identifying quasi-stationary areas in the phase-space: areas where the system is likely to remain confined up to a certain time scale. We call these areas *clusters*.

In order to identify the clusters, the reduced phase-space is split into grid cells, denoted by *G*. The (conditional) transition probabilities are required to group the cells into the sought-for clusters. We capture these probabilities in a transition matrix *T*, whose elements *T*_*ij*_ are defined as the likelihood of going from one grid cell to another within a timelag *τ*, i.e.,

$$\begin{array}{c} {\left(T_{\tau }\right)}_{ij}=\frac{\#\{\left(x_{t}\in G_{i}\right)\wedge \left(x_{t+\tau }\in G_{j}\right)\}}{\#\{x_{t}\in G_{i}\}} \end{array}$$ (1)

Given the phase-space probability density vector ${\vec{\rho }}_{t_{0}}$ (discretized over the grid) at time *t*_0_, one can then calculate the probability density at time *t*_0_ + *τ* by simply operating *T*_*τ*_ on ${\vec{\rho }}_{t_{0}}$:

$$\begin{array}{c} {\vec{\rho }}_{t_{0}+\tau }=T_{\tau }\cdot {\vec{\rho }}_{t_{0}} \end{array}$$ (2)

For small *τ*, realizations would typically move towards a neighboring grid cell or stay within the same one, which results in a sparse matrix with most nonzero elements on the diagonal or slightly off the diagonal, but for longer *τ* realizations would travel further in phase-space. For a given value of *τ*, the matrix elements (*T*_*τ*_)_*ij*_ can then define a ‘transition probability network’ with grid cells as nodes, with the strengths of the corresponding links being determined by the transition probabilities among the grid cells. Clusters can then be identified on this network by searching for groups of nodes (i.e., grid cells) that are strongly *intra*linked (by transition probabilities), but weakly *inter*linked.

In graph theory, there are many clustering methods to achieve this. Here we use the Louvain method [42], which optimizes modularity, defined as the fraction of the edges that fall within the given groups minus the expected fraction if edges were distributed at random (for more details, see [43]). This algorithm merely optimizes the clusters in terms of semi-invariance for a given value of *τ*, but does *not* guarantee it—e.g., it does not guarantee that if the system enters a certain resulting cluster, it will not leave the cluster at a time *τ*′ < *τ*, only to return to it precisely at time *τ*. We will return to this issue in Sec. 5.2.

The resulting clusters, therefore, can be used as good approximations of semi-invariant macro-states of the system, with the caveat that they need to be interpreted in terms of which states of the system they are referring to.

### 2.3 Step 3—Early warnings

Step 3 concerns predicting transitions towards and across macro-states (throughout the rest of this section we will interchangeably refer to them as clusters).

The probability of transitioning from any grid cell *i* to grid cell *j* in time *τ* is given in Eq (1). This can be aggregated by summing over *j* belonging to a given macro-state *k* as

$$\begin{array}{ccc} P_{t_{0}+\tau }\left(\text{to}\;\text{cluster}\;k\right) & = & \sum_{j\in \{\text{cluster}\;k\}}{\vec{\rho }}_{t_{0}+\tau }\left(j\right)\\ & = & \sum_{j\in \{\text{cluster}\;k\}}\left(T_{\tau }\cdot {\vec{\rho }}_{t_{0}}\right)\left(j\right) \end{array}$$ (3)

The result is a first (real-time) indicator of whether we expect a transition towards a cluster. However, being far away from the macro-state hardly ever results in a high (‘alarming’) probability when looking only small time lags *τ*, but it might for a longer time lag. This illustrates that for the construction of an *early warning* metric, one needs to incorporate a variable time lag.

In practice, it is important to know what the minimal lag is, given that one wants to be sure by a certain percentage that the system is remaining in a certain cluster. For example, given that one wants to be sure by a probability of 0.95 that the system remains in cluster *A*, an alarm needs to be given whenever the system enters a cell that allows transitioning towards another cluster *B* with probability ≥ 0.05, and find out what the minimal lag is of doing so. We call this (latter) percentage the *critical probability* *p*_*c*_ and the related minimal lags *τ*_alarm_(*p*_*c*_) (different per grid cell). For the purpose of accurate prediction, a maximum time horizon *t*_max_ is also set depending on the estimated memory of system at hand; meaning that in our calculations, events of entering a cluster at time lags larger than *t*_max_ are not considered in the statistics.

Summarized, a ‘warning’ or alarm is given at any time if the system is likely to enter the cluster at a probability ≥ *p*_*c*_. Attached to this alarm is a time lag *τ*_alarm_ at which this is expected to happen.

### 2.4 Evaluation of predictions

In practice, as well as for the estimation of the parameters *p*_*c*_ and *t*_max_, it is imperative to assess the forecasting skill of the early warning metric. The skill should increase for correct predictions, and reduce in case of false positives (‘false alarms’) and false negatives (‘missed alarms’).

A commonly used metric to assess the accuracy of a forecast is the Brier score [44], defined as:

$$\begin{array}{c} S_{\text{Brier}}=\frac{1}{N}\sum_{i=1}^{N}{\left(p_{f,i}-O_{i}\right)}^{2} \end{array}$$ (4)

where N is the total number of predictions made, *p*_*f*,*i*_ the forecast probability of entering the cluster at instance *i* and *O*_*i*_ is 1 if the system enters the cluster (within *t*_max_), and 0 if not. The Brier score acts like mean squared error of the forecast.

However, the disadvantage of the Brier score is that no variable time lag can be incorporated in it, while the skill should also be penalized by predicting the time lag at which the transition happens wrongly. Therefore, following [18], we use another metric in this study called the Peirce Skill score (PSS), also known as the true skill statistic or as the Hanssen and Kuipers discriminant [45, 46].

To introduce this metric, we first explain different types of correct and incorrect predictions. At every time step *t*, the system is either alarmed (A) or not alarmed ($\bar{\text{A}}$), based on the critical probability *p*_*c*_ and the prediction time *τ*_alarm_ (see Sec. 2.3). We check whether the system indeed entered the specified cluster between now and a specified time from now. If not, we call this a non-occurrence ($\bar{\text{O}}$); but if it does, we call it an occurrence (O) and the time lag at which this happens *τ*_0_ (0 < *τ*_0_ < *τ*_alarm_). The different outcomes are schematically shown in Table 1.

**Table 1:** Various outcomes concerning the correctness of an early warning metric, based on whether an alarms is given (*A*) or not ($\bar{A}$), and whether within the time horizon the macro-state transitions (*O*) or not ($\bar{O}$). Precision in time is assessed by the predicted time lag *τ*_alarm_, the actual time of transitioning *τ*_0_ and a bandwidth *ϵ*. Outcomes are as in the text: various types of false alarms (FA), missed alarms (MA), hits (H) and correct rejections (CR).

	A	A	A	$\bar{\text{A}}$
	*τ*_0_ < *τ*_alarm_ − *ϵ*	|*τ*_0_ − *τ*_alarm_| < *ϵ*	*τ*_0_ > *τ*_alarm_ + *ϵ*	
O	FA2	H	MA2	MA1
$\bar{\text{O}}$	FA1	FA1	FA1	CR

Let us start with the upper row (O), when in reality the system indeed enters the specified cluster. When no alarm is given ($\bar{\text{A}}$), we call this bad prediction a missed alarm type 1 (MA1). Similarly, when an alarm is given, it is only correct if *τ*_alarm_ is close enough to *τ*_0_. We allow for a bandwidth *ϵ* around *τ*_0_; if |*τ*_alarm_ − *τ*_0_| < *ϵ*, the prediction is correct. We call this a hit (H). If the metric predicts it too early (*τ*_0_ < *τ*_alarm_ − *ϵ*), we call it a false alarm type 2 (FA2), and if it is predicted too late (*τ*_0_ > *τ*_alarm_ + *ϵ*), we call it a missed alarm type 2 (MA2).

Next, we consider the lower row ($\bar{\text{O}}$) of Table 1, where the system does not reach the specific cluster within the time horizon (*t*_max_). If an alarm is given (at any *τ*_alarm_), we have a false alarm type 1 (FA1). Similarly, if no alarm is given, then we call it a correct rejection (CR). Later in the paper, we will refer to FA2, MA2 and MA1 as ‘Missed Alarms’ (MA), and to FA1 as ‘False Alarms’ (FA).

With the above, the PSS for a given critical probability *p*_*c*_ is then calculated as follows:

$$\begin{array}{c} S_{\text{Peirce}}\left(p_{c}\right)=\frac{\#\text{H}(p_{c})}{\#\text{O}(p_{c})}-\frac{\#\text{FA1}(p_{c})}{\#\bar{\text{O}}\left(p_{c}\right)}. \end{array}$$ (5)

One sees that the score is rewarded for correct alarms, but penalized for incorrect alarms (including rewarding the score for instances where the early warning metric correctly did not give an alarm).

If *S*_Peirce_(*p*_*c*_) > 0, there is more skill in the prediction than random prediction. Note also that by construction −1 ≤ *S*_Peirce_(*p*_*c*_) ≤ 1; the closer it is to 1, the more skill the prediction has.

## 3 Coupling the framework to data from the Dutch railways

### 3.1 Data description

The data for this study has been provided by the manager of the main railway network in the Netherlands (ProRail), logged at so-called service control points (SCPs). With a total of 801 SCPs spread over the entire network (of which passenger stations are a subset), they divide the Dutch tracks into 1438 smaller segments. Only passenger trains data have been considered, for a number of reasons (as follows). Freight trains are (economically) privacy-sensitive and it is therefore difficult to get a complete dataset. Further, it is only a small fraction of the total railway traffic in the Netherlands: about 5.7% of all Dutch train kilometers in 2017 was by freight trains (numbers courtesy of ProRail). Also, while passenger trains are bound to time schedules and routes, freight trains schedules differ every day. This means that the interaction of freight trains are non-systematic and therefore not really predictable. Moreover, some of the tracks of freight trains are partly separated from passenger trains (like the Dutch ‘Betuweroute’ from Rotterdam to Germany) dedicated to freight trains. This reduces their effect on the dynamics of the whole system even more. We therefore decide to focus only on passenger trains.

We work with the data of one year, from July 1st 2017 to June 30th 2018. The data includes the logging of passing trains, including characteristics of the train, but also the planned time and subsequent delay of the train at 1-second resolution. This data is aggregated to continuous time series on segments at 1-minute resolution. In short, we define delay $d_{i}^{j}\left(t\right)$ of train *j* on segment *i* at time *t* as:

$$\begin{array}{c} d_{i}^{j}\left(t\right)=\{\begin{array}{cc} 0 & \;\text{if}\;t<t_{p}\;\text{(before}\;\text{the}\;\text{planned}\;\text{time)}\\ t-t_{p} & \;\text{if}\;t_{p}<t<t_{r}\;\text{(activity}\;\text{is}\;\text{not}\;\text{yet}\;\text{realized,}\;\text{while}\;\text{it}\;\text{should}\;\text{have}\;\text{been)}\\ 0 & \;\text{if}\;t>t_{r}\;\text{(after}\;\text{the}\;\text{activity}\;\text{was}\;\text{realized)} \end{array} \end{array}$$ (6)

where *t*_*p*_ and *t*_*r*_ are the planned and realized time of an activity of train *j* at segment *i*, respectively. The above definition involves the buildup of delay when a train should be at the segment while it is late, and disappears from that segment when the train exits it, giving rise to a sawtooth pattern of the delay. (Note also that once the delay disappears from a given segment, unless it happens to be the last segment of service for that train, the delay simply continues on the next segment.) We then compute the total delay *d*_*i*_(*t*) on segment *i* at time *t*, by summing $d_{i}^{j}$ over all trains *j* (both directions) as:

$$\begin{array}{ccc} d_{i}\left(t\right) & = & \sum_{j}d_{i}^{j}\left(t\right) \end{array}$$ (7)

For more details, the reader is referred to sections 1 and 2 in S1 Appendix. The aggregation of delay results in 1438 time series spread over the spatial Dutch railway network. Using the notation as in Sec. 2, this means that *M* = 365 ⋅ 1440 = 525600 minutes and *N* = 1438 segments.

### 3.2 Results principal component analysis (Step 1)

The first step concerns the extraction of the most relevant principal components from the data, with ‘relevance’ referring to their suitability to best describe the evolution towards large-scale disruptions.

A commonly used metric for the severeness of delay on the network, is a day-to-day classification defined by ProRail. All days are labeled with one of four (severity) categories: green, neutral, red or black. These classes are based on the punctuality and cancellations of trains on important train tracks. ‘Green’ (46 out of 365 days) days refer to ‘quiet days’: few canceled and delayed trains, while ‘red’ (21) and ‘black’ (6) days refer to ‘disrupted days’, that contain a lot of delays and cancellations. ‘Neutral’ (292) days are those that are neither quiet, nor disrupted days. We term the green and neutral days together as ‘regular days’. For more details, the reader is referred to section 3 in S1 Appendix.

To get the relevant PCs, we choose to perform PCA only on the delay data from the disrupted days (‘red’ or ‘black’), as by definition they explain the most variance for disrupted situations (a robustness check on the PCA results is performed in S2 Appendix). To ascertain the relevance of these PCs, we have assessed the performance of the PCs on the entire dataset (i.e., including delay data from the regular days): for example, if the PCs calculated from the delay data on disrupted days barely explain any variance on regular days, they may not be useful for analyzing the evolution of the system towards disrupted states (in that case, they would essentially only explain persistent patterns on the disrupted days).

Subsequently, the question arises regarding which and how many PCs we need to retain to construct the reduced phase-space. The metrics we use to answer these questions are the explained variance by, and persistence in time of, the PCs. The latter we define as the timescale *τ*_0_ at which the autocorrelation function decays, which we extract by fitting a function $e^{-\tau /\tau_{0}}$ to the autocorrelation function of the PC amplitudes, scaled to its value at zero time-lag. From the combined delay data, it turns out that the first two PCs distinguish themselves from the rest (more details in S1 Appendix). This motivates our choice to retain only PC1 and PC2 to define the reduced (two-dimensional) phase-space for describing the dynamics of the Dutch railway system.

Putting all the above ingredients together, our results for step 1 are as follows. The variance explained on the disrupted days by the first two PCs are 16.0% and 9.5%, respectively, while these numbers change to 13.0% and 16.0%, respectively when the variance is calculated on the full dataset, i.e., including regular days. Sorting the PCs by variance explained makes sure that we use those components that show more-or-less robust covariant patterns, rather than the effect of (incidental) individual cases.

**Fig 2:**
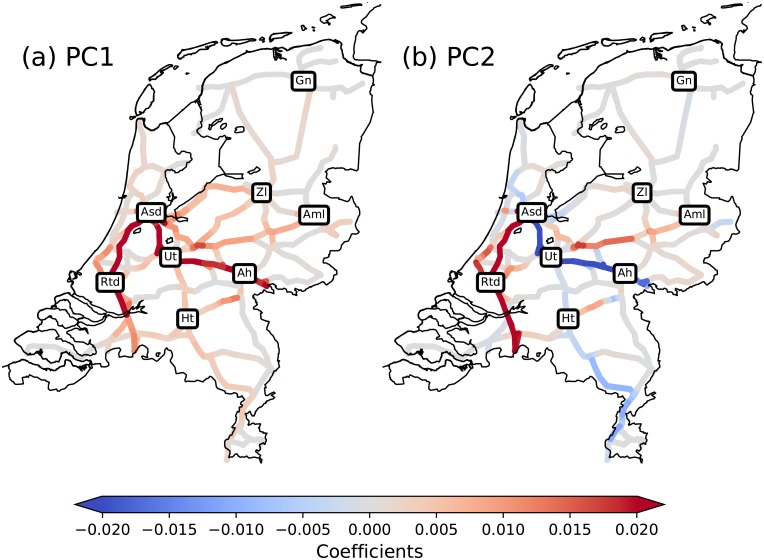
The first two relevant EOFs for the Dutch railway system, explaining respectively 13% and 16% of the variance over a full year. A running spatial average-smoothening is applied for visualization reasons. Abbreviations refer to important passenger stations: Amsterdam Central (Asd), Rotterdam Central (Rtd), Utrecht Central (Ut), Arnhem (Ah), Groningen (Gn), ’s-Hertogenbosch (Ht), Amersfoort (Amf) and Almelo (Aml).

**Fig 3:**
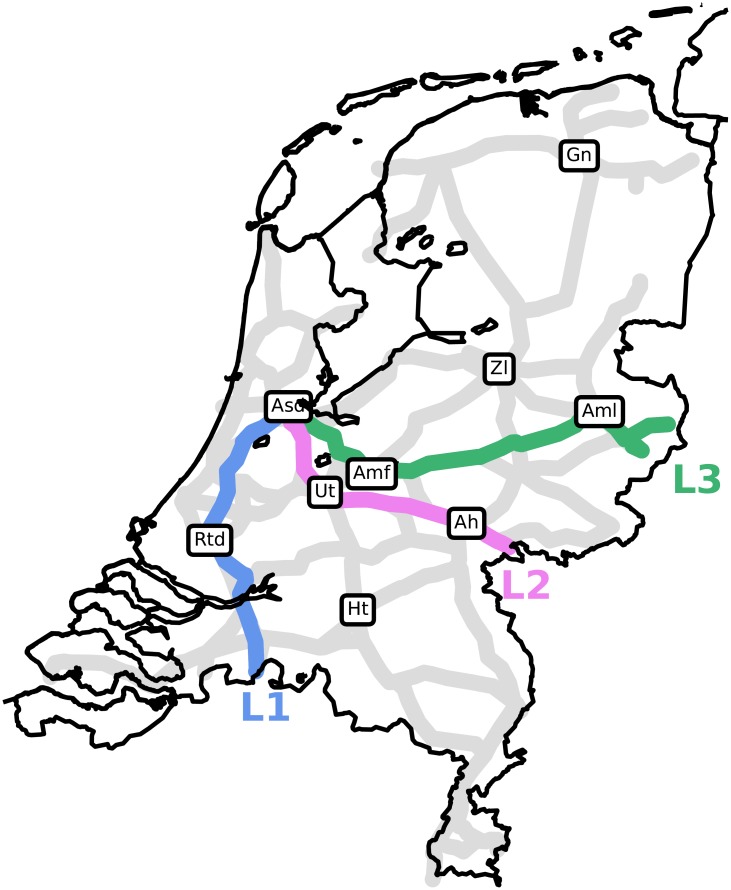
Three important lines L1, L2 and L3. Abbreviations as in Fig 2.

The corresponding EOFs, i.e., the spatial plots of the individual PC elements are shown in Fig 2. Segments that have high equally-signed amplitudes in these plots are parts of the network at which delay often co-occurs. Three dominating train lines can be distinguished in Fig 2 (see Fig 3):

L1: The line from Amsterdam southward through Rotterdam. It is connected to Belgium by trains ultimately reaching Antwerp and Brussels.
L2: The line from Amsterdam southeastward towards the cities of Utrecht and Arnhem. It continues towards the German cities of Düsseldorf, Köln and Frankfurt.
L3: The line from Amsterdam eastward towards the cities of Amersfoort and Almelo. This line is connected to major German cities like Münster, Dortmund and Berlin.

These lines do not only incorporate the effect of long-distance (international) trains that are prone to building up delay, but they also include busy tracks and major cities in the Netherlands, explaining the large amount of variance on these lines.

**Fig 4:**
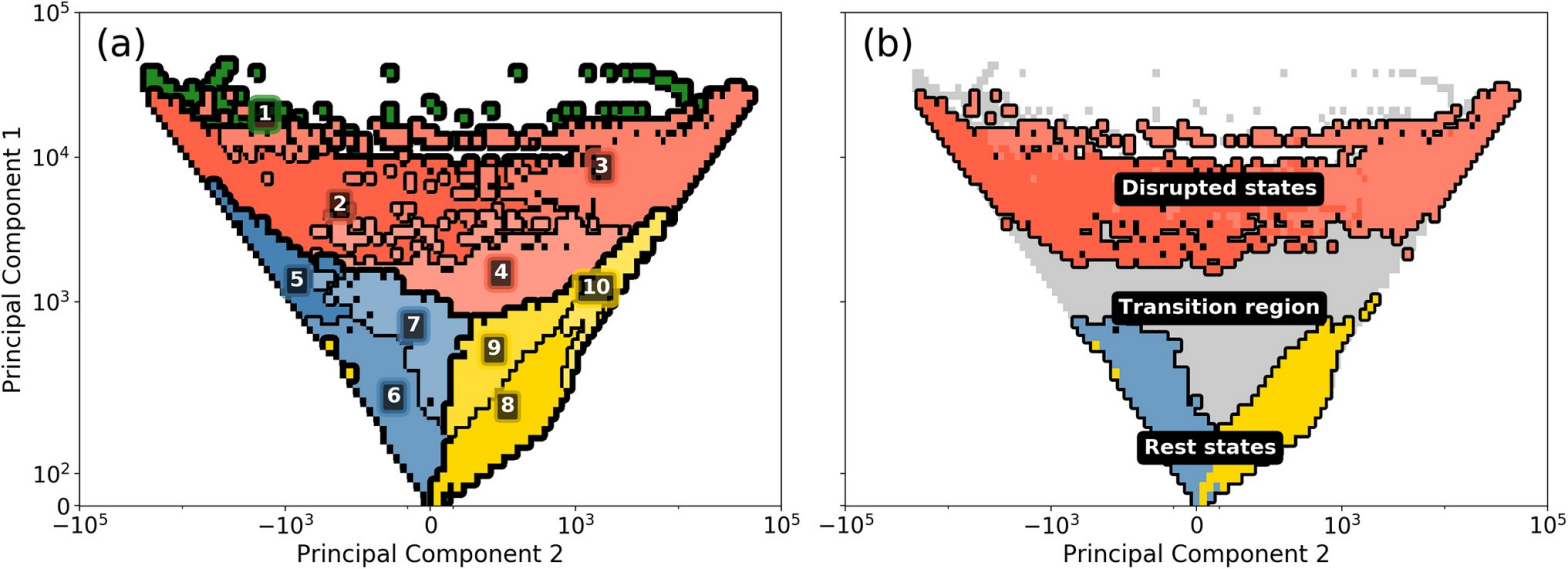
Phase-spaces showing (a) clusters in colors, and subclusters in various shades of the same color, and (b) how we interpret various parts of the phase-space: Rest states (subclusters 6 and 8), transition regions (4, 5, 7, 9 and 10) and disrupted states (2 and 3). Colors have been used only for those areas, where there are realizations. The choice of colors is arbitrary and has no relation to the classes defined in Table A of S1 Appendix.

We can interpret the PCs in terms of the delay spread across these three lines, using the EOFs in Fig 3. It is important to stress that delay is almost always a positive variable on any segment. A negative value anywhere at any time means that the sum of the delay of all planned trains at that time and track is negative. Trains may be early, but such occurrences are infrequent and not by a large amount of time. Note that this statement does not contradict the plots in Fig 4, since the actual delay on the network at any given time linearly relates to the amplitudes of these PCs, which can be both positive and negative. However, as is visible in Fig 2a, almost all coefficients of PC1 are positive, resulting in only positive PC1 amplitudes. Looking at the coefficients of PC2 in Fig 2b, we see (large) negative coefficients on L2 and positive coefficients on L1 and L3.

### 3.3 Results identification of macro-states (Step 2)

Within the PC1-PC2 reduced phase-space (we henceforth refer to it simply as ‘phase-space’ for brevity) we now identify states, a process that we describe in this section.

First, we compute the region of the phase-space covered by the entire year’s delay data, obtained simply as the amplitudes of the two PCs (*M* data points all together). This region exclusively corresponds to PC1 ≥ 0. We then discretize this region into 123 × 123 grid cells on a logarithmic scale, on which we compute the transition matrix *T*_*τ*_ with *τ* = 30 minutes. We follow this up by performing Louvain clustering on the transition matrix data, leading to the identification of four clusters that are shown in Fig 4a in thick black boundaries. (Robustness of the Louvain clustering against the choice of grid resolution and *τ* has been checked in section 2 of S2 Appendix).

We find that each cluster in Fig 4a, being so large, contains multiple delay configurations of the full system. In order to differentiate among these, we perform Louvain clustering a second time within each cluster. This action leads to the identification of further clusters within each cluster; we call these *subclusters*. Our calculation reveals the existence of ten subclusters all together, numbered 1 through 10; these are shown in different colors and thin black boundaries in Fig 4a.

We next interpret the subclusters in terms of macro-states they may represent. Obviously, the area around (PC1,PC2) = (0,0)—i.e., the origin—must represent the so-called rest states, since in this area the delays are small. (Indeed, every day at the beginning and at the end of service the system respectively starts from and returns to the origin.)

The interpretation of the subclusters as macro-states is more subtle, and can only be ascertained by evaluating all instances of the system within each subcluster. To this end, we analyze each instance in terms of two variables: (a) amount of delay, and (b) in what respect various day-classes (as in Table A of S1 Appendix) are represented, by defining a ‘bias factor’ *B* as

$$\begin{array}{c} B\left(i,j\right)=\frac{P(i|j)}{P(i)} \end{array}$$ (8)

where *P*(*i*) is the probability of a realization to have day-label *i* (i.e., green, neutral, red or black), while *P*(*i*|*j*) is the probability of a realization to have the same label within subcluster *j*. We add the day-labels in this analysis since delay alone does not signify the severeness of disruption—e.g., a sharp spike in delay is not necessarily a severe event if it is resolved quickly. Also (as noted above), every day the system starts from (PC1,PC2) = (0,0); for this reason, even the black days have some realizations in the subclusters around (0,0).

The bias factor reflects the prevalence of a certain day-label within each subcluster in comparison to its overall prevalence. For example, if *B*_*G*_(3) is less than 1, it means that green days are less frequent in subcluster 3 (of all subclusters considered). The results are shown in Table 2.

**Table 2:** Biases of realizations of all labels per (sub)cluster. Lower-script characters of *B* refer to the bias factor on ‘green’ (*G*), ‘neutral’ (*N*), ‘red’ (*R*) and ‘black’ (*B*) labeled days, as defined in Table A of S1 Appendix. The delay in the last column depicts the summed delay (in hours) over the whole network (i.e., summed over all segments) averaged over the realizations that are inside the respective (sub)clusters.

(Sub)cluster	*B*_*G*_	*B*_*N*_	*B*_*R*_	*B*_*B*_	Delay
1	0	0.33	1.45	39.5	95.6
2-4	0.40	0.98	2.16	2.42	20.2
2	0.56	0.91	2.57	3.31	23.3
3	0.34	0.98	1.69	4.22	33.2
4	0.36	1.01	2.20	1.32	13.4
5-7	1.21	0.99	0.84	0.65	3.9
5	1.01	1.01	1.04	0.46	8.0
6	1.44	0.96	0.62	0.68	2.2
7	0.77	1.02	1.29	0.6	6.6
8-10	1.03	1.02	0.72	0.68	3.8
8	1.25	0.99	0.68	0.76	1.4
9	0.73	1.06	0.88	0.46	6.3
10	0.74	1.07	0.54	0.80	8.9

Using the numbers shown in Table 2, we provide an interpretation to the subclusters shown in Fig 4b. The interpretation of the phase-space is in thee parts:

- Subclusters 6 and 8 have the highest *B*_*G*_ scores, indicating a strong bias on green days to be in these subclusters. Moreover, they have relatively low bias scores on other days, and by far the lowest total delays. These are characteristics of the system being ‘at rest’ and therefore we use these subclusters as an approximation of the *rest state*. This choice is also supported by the fact that these subclusters are closest to the origin: obviously PC1 and PC2 are low in magnitude when delays are small.
- The largest *B*_*R*_ and *B*_*B*_ scores and the largest total delays are found in subclusters 1 to 4. Only 0.14% of all realizations are inside subcluster 1, which accounts for only about 12 hours in the entire year. (Because of this data sparsity, we will not consider subcluster 1 separately). While subclusters 2 and 3 separate themselves from subclusters 5 to 10, subcluster 4 seems to be somewhat in between: it has a *B*_*N*_ score of above 1, meaning that neutral days are above averagely represented in this subcluster. Moreover, its total delay (13.4 h) is on average only slightly higher than the total delay in some of the lower subclusters [e.g., 5 (8.0 h) and 10 (8.9 h)]. We therefore choose to denote subclusters 2 and 3 as *disrupted states*. (However, when presenting the early warning results in following sections, we will include subcluster 4 for completeness.)
- The rest of the subclusters (4, 5, 7, 9 and 10) then automatically form a *transition region* between the rest and the disrupted states. Computation of conditional probabilities (not shown) of reaching one subcluster from another reveals that subclusters 5 and 10 act as transition regions for the system to move towards subclusters 2 and 3, while subclusters 7 and 9 have a lot of dynamical exchanges with subcluster 4, which in turn acts as a conduit for the system to enter subclusters 2 and 3.

**Fig 5:**
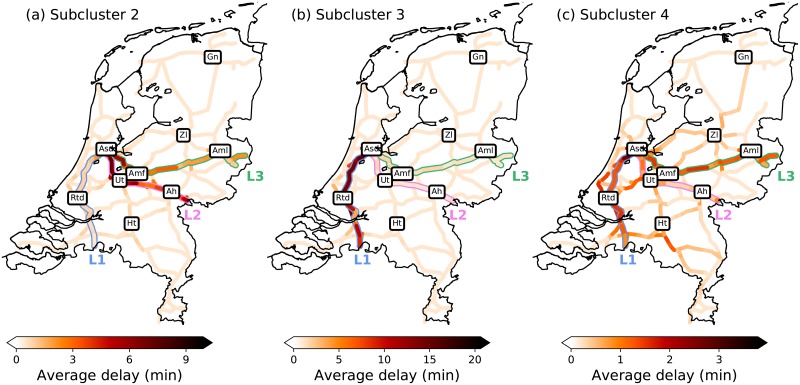
The color of each segment shows the average delay of all realizations in subclusters (a) 2, (b) 3 and (c) 4 on the particular segment. Abbreviations as in Fig 2. The lines L1, L2 and L3 are highlighted.

From these, we conclude that the subclusters 2 and 3 are the ones of interest for studying evolution towards disrupted states. In order to get an intuition on which delay configurations of the full system they represent, the average delay per segment per subcluster is shown in Fig 5 (for the sake of completeness, we also add subcluster 4). The delays seem small (∼ 2-20 minutes on important trajectories), but note that the presented results are averages over all realizations per subcluster: there are many types of delay combinations on these important trajectories, which make the signal per subcluster appear diffuse. The delay patterns in Fig 5 are quite distinct from each other, and indeed allow for intuitive interpretations of the three subclusters: an upper-left region with delays as L2 and L3 (subcluster 2), an upper-right region with delays on L1 (subcluster 3), and a middle region containing less severe and more spread out delays (subcluster 4).

As the subclusters are distinctively positioned in the phase-space, we could have qualitatively anticipated the above results already from Fig 2. The fact that Fig 5 confirms these patterns reflects that the delay signals in the *reduced* phase-space indeed corresponds well to the delay signals in the actual realizations. Moreover, we see in Fig 5 that the magnitude of the average delay patterns differ (note the color bars). The fact that the maximum average delay in subcluster 3 is much higher than in subcluster 2 and 4 does not necessarily mean that the delays in general are longer (although that it is the case on average can be seen in Table 2); it could also mean that the delays are more consistent with where on the network they occur. In particular, one could conclude that delays in subcluster 3 are longer and more concentrated on the network.

### 3.4 Results early warning and skill score (Step 3)

Using subclusters 2 and 3 as approximations of two different types of disrupted macro-states, the prediction towards them amounts to predicting a transition towards large-scale disruptions. That is the aim of the third step.

As described in section 2.3, the final strength of our prediction is dependent on the choice of parameters *p*_*c*_, *ϵ* and *t*_max_. First, there is an optimum in the skill with varying critical probability *p*_*c*_: a *p*_*c*_ that is too low increases the amount of FA1, but a *p*_*c*_ that is too high increases the amount of MA1 (or decreases H). Second, the skill is dependent on the bandwidth *ϵ*. The skill grows for larger *ϵ*, but that also means loss of accuracy in the timing of the predicted event. It is therefore a trade-off between accuracy in the time-bandwidth and accuracy in being right in predicting. The third parameter that influences the skill is the time horizon *t*_max_. If *t*_max_ is small, the overall skill will generally increase, simply because it is easier to predict events that are imminent. Here too there is a trade-off: a small time horizon means only short-term predictions, which reduces the value of giving a warning, as there would then be little time left to prepare for taking intervening measures.

Considering the trade-off for all the involved parameters, the ideal situation would be a high skill, at a small *ϵ* (at least $\epsilon ⪡{\bar{\tau }}_{\text{alarm}}$, where ${\bar{\tau }}_{\text{alarm}}$ is the average alarm lag for correct alarms), a large *t*_max_ and a large *p*_*c*_. Nevertheless, a good choice of the parameters *ϵ*, *p*_*c*_ and *t*_max_ is important, because it modulates the skill greatly. The right choice will depend on the system and what type of accuracy the user deems important. See section 4 in S2 Appendix for the parameter sensitivities of predicting evolution towards various subclusters.

**Fig 6:**
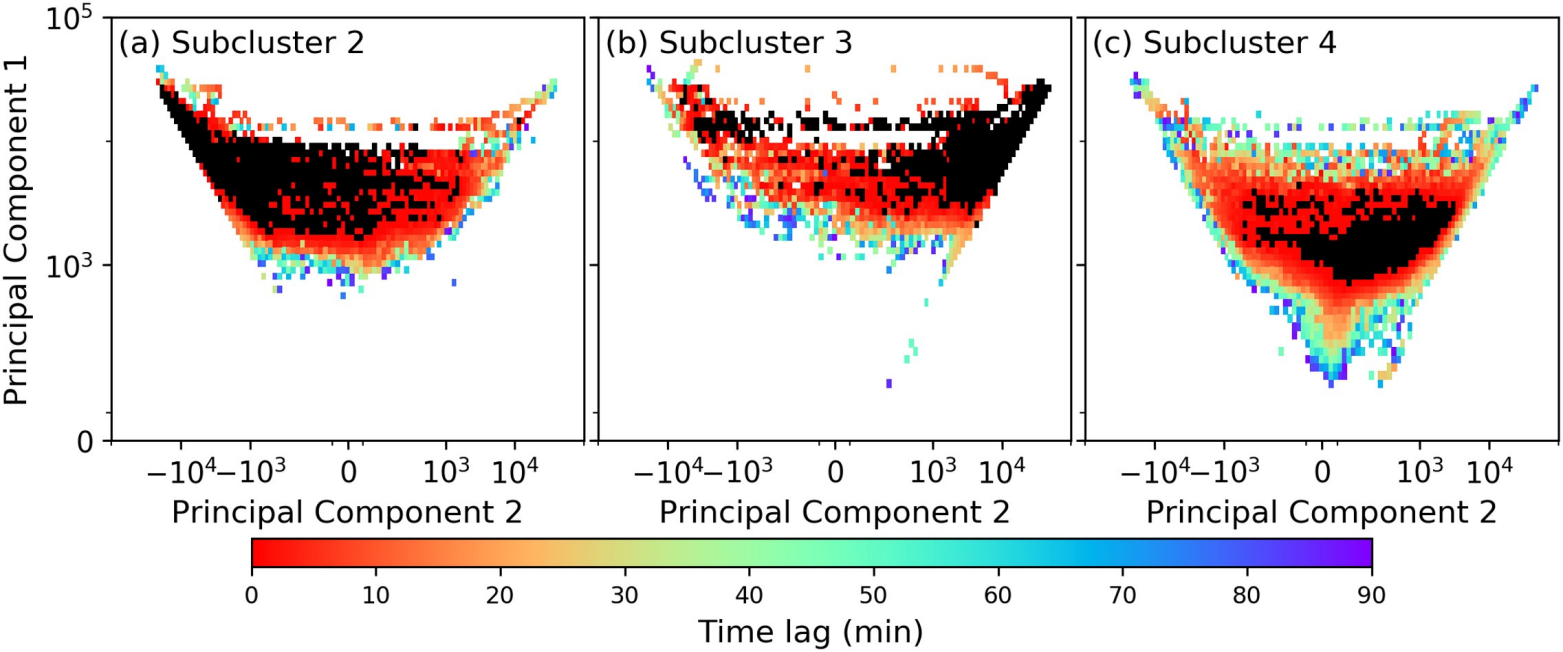
Phase-space showing (in black) (a) subcluster 2, (b) subcluster 3 and (c) subcluster 4, and the predicted time lag *τ*_alarm_ (in colors) for entering these subclusters for *p*_*c*_ = 0.08; *τ*_alarm_ values are discretized to 5 minute intervals. No color (white) means no alarm is given from these grid cells, reflecting a time horizon *t*_max_ of 90 minutes.

For *p*_*c*_ = 0.08 and *t*_max_ = 90 minutes, the time lags at which an alarm is given are shown in Fig 6 (i.e., for every grid cell, we determine whether there is a probability of at least 0.1 to reach the specific subcluster at a time lag ≤ 90 minutes). In Fig 6a we see that alarms are mostly given in the upper part of the phase-space: a total of 60% of the grid cells representing actual realizations is covered. Most alarms are given at low time lags (i.e., close to the subcluster). More long-range predictions can be made mostly in the upper-right corner and in subclusters 5 and 7 (cf. Fig 4a), even up to 90 minutes. The large colored ‘tail’ in the upper right of the figure reflects the possibility of transition between an L1-dominated delay signal (Fig 5b) towards an L2/L3-dominated delay signal (Fig 5a), possibly through propagation of delay via Amsterdam, the city that connects the three lines.

The time lags for subcluster 3 are shown in Fig 6b. The number of colored grid cells is about the same as those in Fig 4a: 58% of the grid cells representing actual realizations is covered, reflecting alarms at time lags ∼30 minute of prediction in subclusters 10 and 3. Further, alarms are given only in the upper part of the phase-space. From Fig 6a and 6b we also see that there are a lot of system evolutions from subcluster 2 towards subcluster 3 (and *vice versa*).

For completeness, we also calculate the time-lag results for subcluster 4 (although this does not belong to the disrupted state defined in the previous subsection). The results are shown in Fig 6c. As expected, it has a much more spread-out pattern of *τ*_alarm_ as 79% of the grid cells representing actual realizations is covered. This confirms our choice to interpret it as a transition region (cf. Fig 4b): this subcluster shares boundaries with many others, and therefore easily visited by the system. The predicted times are also not mainly restricted to short lags (a more pronounced signal is visible for values between 60 and 90 minutes). Revisiting Fig 4, one sees that almost no alarms are given from subclusters 6 and 8, which points to the fact that subclusters 7 and 9 (5 and 10 in a different manner) act as ‘buffer regions’, from which the system can quickly recover (refraining from transitioning towards the disrupted region).

The corresponding early warning skills, for *p*_*c*_ = 0.08, *t*_max_ = 90 and *ϵ* = 30 minutes, are shown in Table 3. A first observation is the difference between subcluster 4 and (each of) subclusters 2 and 3: the system is inside subcluster 4 more often in general (2.3 h per day), as well as more often on relatively ‘quiet’ days (green and neutral). This may refer to the fact the combined lines L1, L2 and L3 are often delayed together (such that PC2 amplitude is neither strongly positive nor strongly negative, and PC1 amplitude is relatively large, cf. Fig 2). Further, subclusters 2 and 3 are most often reached on red and black days, coinciding with higher values for H and O.

**Table 3:** Average amount of hours per day that the prediction system records a Hit (H), occurrence (O) or that the system is inside the subcluster (I) for variable severity label (for example: A value of 0.4 at row I, subcluster 2 and column G means that on average, 0.4 hours of green days are inside subcluster 2). We also present the Peirce Skill score (PSS) for each subcluster and days. Capital characters in header refer to green (G), neutral (N), red (R) and black (B) days. Parameter settings: *p*_*c*_ = 0.08, *ϵ* = 30 minutes and *t*_max_ = 90 minutes.

Subcluster	Variable	G	N	R	B	Overall
2	I	0.4	0.7	2.1	2.8	0.8
	H	0.4	0.8	2.3	3.0	0.6
	O	0.9	1.5	3.5	4.0	1.6
	PSS	0.15	0.19	0.38	0.39	0.2
3	I	0.3	0.9	1.5	3.5	0.9
	H	0.3	0.6	1.3	2.6	0.6
	O	0.6	1.3	2.6	4.0	1.3
	PSS	0.08	0.17	0.21	0.36	0.16
4	I	0.8	2.3	5.1	3.0	2.3
	H	2.0	3.6	5.3	4.2	3.5
	O	2.5	4.0	5.8	4.7	4.0
	PSS	0.25	0.32	0.38	0.47	0.32

Concerning the PSS in Table 3, we see that the system evolution towards subclusters 2-4 are the most difficult to predict on green days, while it is far easier to do so on red and black days. On green days, these subclusters are visited intermittently and in a less structural manner, which makes predictions more difficult. The PSS scores for subclusters 2 and 3 are on average roughly equal, but with a strong difference on red days (0.38 to subcluster 2 versus 0.21 to subcluster 3). The average PSS for subcluster 4 is much higher than those for subclusters 2 and 3. In general, for these parameter settings the skill scores are roughly around 0.1-0.3. These values are larger than 0, indicating (a) that there is skill in our predictions, and further (b) that our framework could potentially anticipate approaches towards disrupted macro-states.

## 4 Implications for day-to-day operations: Two case studies

To showcase the potential strength (and limits) of our framework for anticipating approaches towards disrupted macro-states, we analyze its performance on two specific days as case studies (Wednesday January 3rd 2018 and Thursday April 19th 2018). The case studies are chosen based on the fact that these are ‘red days’ on which the system indeed evolved towards the disrupted state (cf. Fig 4). Note that if the system would not enter the disrupted state, the performance would be calculated only on an empty-occurrence (O) set, resulting in *S*_*Peirce*_ ≤ 0 *by definition*. Indeed, if we are interested in the performance of the predictions on sets with no occurrences, some other skill score could be a better choice. See S3 Appendix for a discussion on a regular day, on which the system does not enter the disrupted state.

We first provide an overview of the statistics for these days in Table 4. Therein, we see—upon comparing to the total delay and amount of cancellations (per minute) over the entire year—that both January 3rd and April 27th have strikingly different characteristics than the system has on average. The average total delay of 5.5 hours on average days can be interpreted as 13.8 seconds delay on *every* segment at every instance of the day. Both days contain 2-3 times more average total delay, and the maximum total delay on January 3rd is even about 4 times as high as on average. The amount of canceled train activities per minute is also larger, with slightly higher amounts on April 19th than on January 3rd.

**Table 4:** Two case studies in comparison to the statistics over the entire year. Here, we use *p*_*c*_ = 0.08, *t*_max_ = 90 minutes and *ϵ* = 30 minutes for the calculation of the predictability (Peirce Skill score, or PSS). Cancellations mean canceled train activities per minute. A running mean of 30 minutes has been applied. See text for details. The alarm lag time *t*_alarm_ values are shown only for successful alarms (‘Hits’). Statistics for all days are given in median values.

Variable	Metric	All	03/01	19/04
Delay	Average	5.5 h	17.3 h	13.5 h
	Maximum	23.4 h	101.4 h	35.6 h
Cancellations	Average	2.1	6.2	6.6
	Maximum	7.7	17.0	22.2
Predictions to 2	PSS	0.20	0.37	0.31
	Max *t*_alarm_	90 min	87 min	50 min
	FA%	8.9%	12.8%	21.0%
Predictions to 3	PSS	0.16	0.34	0.14
	Max *t*_alarm_	89 min	65 min	86 min
	FA%	7.0%	5.2%	17.7%
Predictions to 4	PSS	0.32	0.34	0.45
	Max *t*_alarm_	90 min	82 min	75 min
	FA%	35.6%	39.7%	35.6%

The overall score might give an indication of the predictability for individual subclusters, suggesting that the movement towards subcluster 4 is easier to predict. This is however not the case *per se*. As explained above, an issue with the Peirce Skill Score is that for specific subclusters the score is purely negative on days when the subcluster is not reached. This leaves a bias in the skill score: independent of their predictability, subclusters that are more often reached generally have a higher PSS (we have seen earlier that subcluster 4 is more often visited).

Another important observation in Table 4 is the number of false alarms. Although the metric does penalize false alarms, a small amount of correctly predicted occurrences can result in a high PSS, even when the number of false alarms is also large. Considering that for railway companies, false alarms can in practice be more destructive than missed alarms, it is important to keep track of the false alarm rate as well. Note that the false alarm rate involving subcluster 4 is quite high, which indeed points to the fact that it is easily visited and not *per se* related to large disruptions. In comparison, the false alarm rates towards subcluster 2 and 3 are much less.

### 4.1 Wednesday January 3rd 2018

**Fig 7:**
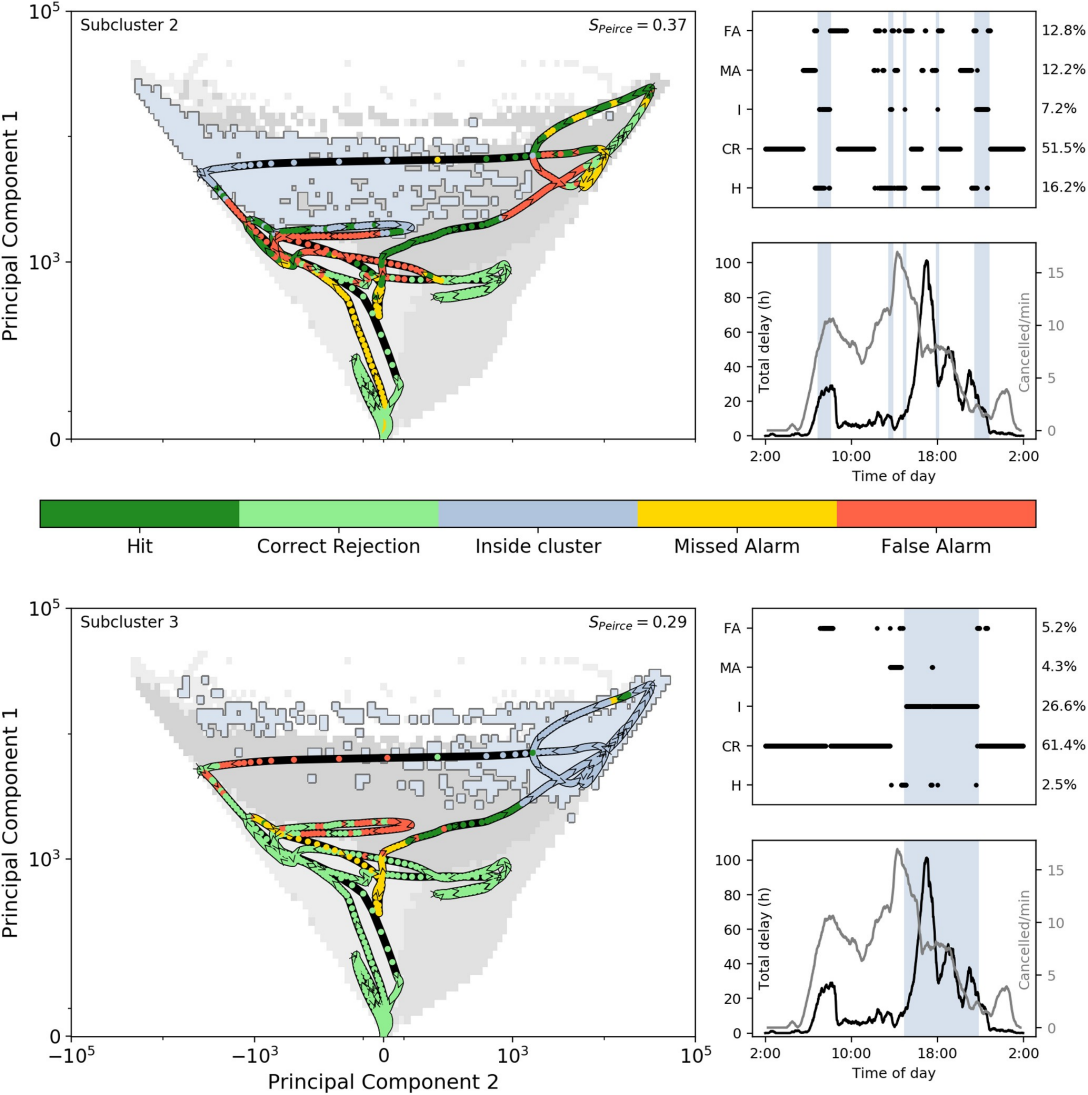
Evolution of the system in the phase space on 3rd January 2018 in dots (V-shaped arrows show the direction). Dot coloring shows the predictions to (a) subcluster 2 and (b) subcluster 3, as shown by the color bar at the bottom: ‘Hit’ indicates a correct prediction to enter subcluster, ‘Correct rejection’ means the scheme was correct not to give an alarm, ‘False alarm’ indicates an incorrect alarm due to no entrance of the disrupted subcluster and ‘Missed alarm’ means false negative (alarm should have been given). Subclusters 2 and 3 are marked in light blue (other clusters in various gray shades). On the right, for each cluster the different outcomes in time are stated including percentages and overall score (top) and the total delay (black) and canceled activities per minute (Gray) is shown (bottom). The vertical blue-colored columns indicate periods of time inside the subcluster. Parameters used: *p*_*c*_ = 0.08, *t*_max_ = 90 min and *ϵ* = 30 min.

Fig 7 shows the system’s trajectory in the phase-space on this day along with all prediction outcomes in the colors of the dots, and the corresponding evolution of total delay and cancellations per minute (see panels on the right).

Since, by construction, the system is at (0,0) in the phase-space at the start and at the end of each service day, the system is seen to be around (0,0) at early morning and late night. This coincides with a lot of correct rejections. However, already around 6:00 hrs, a strong increase in delay forces the system into subcluster 2, and it is predicted only shortly before (visible in the first blue shaded area in the upper-right panels of Fig 7a). This event moves the system’s position in the phase-space to the upper-left, corresponding to delays in the west of the Netherlands (lines L1 and L3). During this period, some false alarms towards subcluster 3 are also given. After this event, the system returns to ‘safer areas’ in the phase-space, resulting in correct rejections for both figures around 10:00 hrs. After this, delay builds up strongly towards the upper-right of the phase-space, relating to delay in the center of the Netherlands, on line L2. This corresponds to the long period (large part of the afternoon and evening) that the system remains within subcluster 3, keeping the total delay to also remain high for a long time. During this period, some small entrances of subcluster 2 are recorded, but this is due to the existence of disconnected subcluster 2 cells scattered within subcluster 3. The system leaves subcluster 3 to return to subcluster 2 again for another hour, after which the system slowly moves back towards the origin.

Correct predictions towards subcluster 2 are made up to 87 min beforehand. We can see in the right panels that not all occurrences are predicted equally well—the entrance of the subcluster in the early morning, for example, is not predicted far ahead. Considering the prediction towards subcluster 3, there was only one time the system entered the subcluster (before it remained there for quite some time), which is predicted roughly half-an-hour before, and on one instance there was a correct prediction at 65 min before.

The right panels of Fig 7 also show the evolution of the total delay and cancellations. For cancellations we use the amount of train activities (departure, arrival, short stops etc.) that were scheduled, but canceled, per minute. This is a measure of the reduction of ‘stress’ on the system by human decision. Note that all large delay spikes are situated in either of the two subclusters, reflecting that these subclusters do indeed refer to a disrupted state. The first canceled activities started in the early morning already, reflecting the early start of the problems on the network.

### 4.2 Thursday April 19th 2018

**Fig 8:**
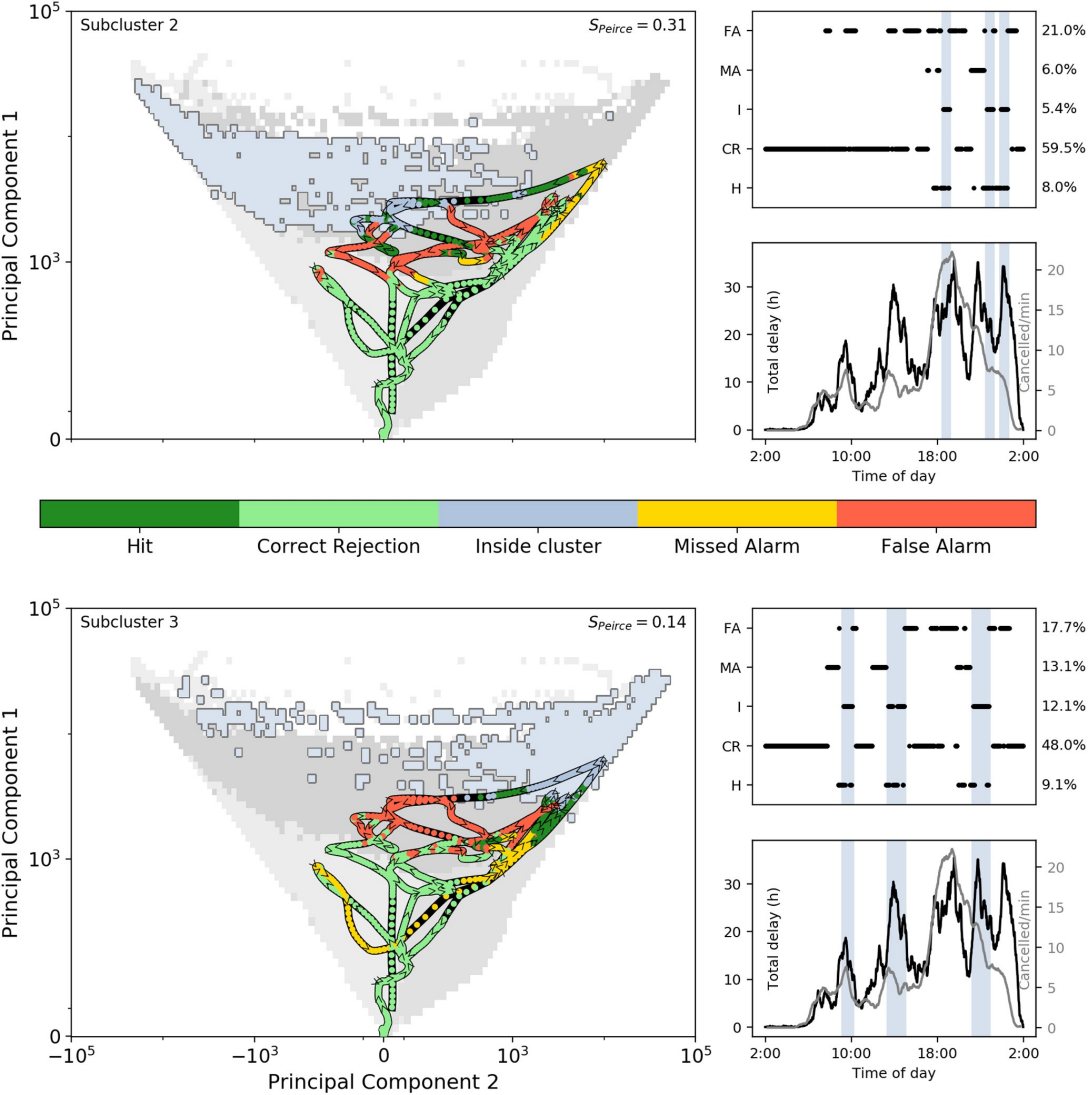
Same as in Fig 7, but for the case of April 19th 2018.

Similarly, Fig 8 shows the system’s dynamics in the phase-space for April 19th 2018. On this day, the system mostly remains in the positive-PC2 part of the phase-space. Subcluster 2 is only reached on a few short instances, and the system never reaches the core of the subcluster (merely visiting the disconnected scattered cells belonging to it). We focus our analysis below on the instances that it reaches subcluster 3: three instances in total.

The first instance (around 9:00-10:00 hrs) seems difficult to predict (correct predictions are made only up to 35 minutes in advance), resulting in several missed alarms. This event coincides with a small but clear peak in the total delay and cancellations.

The second instance (around 14:00 hrs) is better predicted, but there are still some missed alarms visible. Correspondingly, we see that this coincides with a strong increase in total delay, concentrated on L1 and L3. The system remains rather delayed after this.

The third instance is in the late evening (around 23:00 hrs) and does coincide again with a strong peak in total delay, but prior to that there is another peak. This peak actually does not correspond to an entry to subcluster 3, but to (deep inside) subcluster 4. The third instance is however better predicted, up to 82 minutes in advance. The ‘quality of prediction’ is remarkable, as this event happens to be one of the strongest amplification events in the data, tripling the amount of total delay within about an hour.

Overall, the correct predictions are mainly confined to a certain part of the phase-space as most of the ‘Hits’ are found when the system is in subcluster 10. A lot of false alarms are given when the system is in subcluster 4. Missed alarms can be found far away from the subcluster of interest (mostly in the lower-left part of the phase-space), which makes sense as it is rare that the system moves so quickly from that part of the phase-space all the way towards subcluster 3.

We can distinguish five delay peaks in the panels on the right in Fig 8; these are indeed the instances in which the system is inside subcluster 2 or 3.

## 5 Discussion and conclusions

### 5.1 Summary

For systems whose dynamics are poorly known or strongly heterogeneous (like in many socio-technical systems) we have developed a framework to identify macro-states, and further analyze and predict transitions across them. We have coupled the framework to a year’s data from the Dutch railways, and predict large-scale disruptions.

The framework consists of three steps. The first step consists of a dimensional reduction, based on identifying the relevant patterns to define a reduced phase-space, which, for the railways, has been achieved by a principal component analysis. This choice to determine the relevant patterns is based on both the amount of variance explained, as well as the persistence of the patterns in time, defined by the time-scale for the autocorrelation functions of the principal components. For the Dutch railways, we have found that the first two principal components are the most relevant. These components reflect a combined signal of three important international railway lines.

The second step consists of defining macro-states as the (quasi-invariant sub)clusters in the reduced phase-space. We have achieved this by splitting the phase-space into grid cells, calculating the transition matrix with elements consisting of conditional probabilities of transitions between cells, and further applying a clustering algorithm. For the Dutch railways we have found 10 subclusters, which we have divided among ‘rest states’, ‘transition regions’ and ‘disrupted states’ upon analyzing the realizations of the system within these subclusters. The average delay patterns per subcluster has led us to distinguish various types of disruptions—one focused on the line from Amsterdam southward via Rotterdam to Belgium, another on the lines from Amsterdam to Germany, and a third showing a combination of other patterns.

The third and final step consists of the prediction of entering specific subclusters. For the Dutch railways, the subclusters of interest correspond to disrupted states. Using conditional probabilities obtained from the transition matrix, we have devised an early warning procedure that, given a certain threshold probability, gives an alarm at a certain time lag. The skill of the alarm procedure has been analyzed using the Peirce Skill score. Applying this to the Dutch railway system, we have found reasonable Peirce Skill score towards disrupted states. This reflects the potential of this framework to anticipate macro-state transitions towards disrupted states.

### 5.2 Discussion

Several aspects of our framework need to be discussed. First and foremost, for railways the delays are a combined result of (among other factors) (a) the physical interactions of trains and infrastructure (e.g. a broken train blocking a piece of track), (b) accidents and sheer coincidences (e.g. illness of crew, broken switches or trees falling on tracks), and (c) human influence on the system (passengers, crew, traffic controllers and dispatchers). All these factors cause non-systematic noise in the system. For example, the specific nature and duration of a problem involving a switch in the tracks largely determines its effect on delay, and although there are protocols bordering human decision making (at various levels) in certain circumstances, in practice every situation, person’s reaction and their combined effect on the evolution of delay is unique in every situation. In a data-driven framework, all these factors (both systematic and non-systematic) are intertwined, and there is no way to disentangle them. Although this increases the uncertainty, it can be considered a strength of this analysis to not ignore the human impact on the system—after all, human influence is an integral part of the dynamics of the system. Many studies only focus on element (a), micro-simulating only train interactions, which results in an uncertainty in itself when it comes to prediction of macro-scale delay.

Related to the above is the fact that in many ST systems like railways and disease spreading, human control elements (interventions) play a role specifically in the case of macro-state transitions, both to prevent such events or to recover from such events. Especially in railways, it is difficult to distinguish whether control measures have a damping effect on the disruption’s spread, or (although necessary in case of disruption) play an amplification role in spreading the disruption. In part, these control elements are correctly incorporated in the transition matrix and clustering: areas in the phase-space where the system is often stabilized (preventing amplification) can be seen as areas that are relatively safe from transitioning. However, it remains difficult to disentangle the cause versus the effect of control measures. They are inseparable from the data used in this study, exert changes on a spatial micro scale rather the macro scale used in this study, and contain discrete and sparse data that is hard to couple to the definition of the dynamic delay variable. More research is needed to develop methods that find the ‘laws’ underlying the system’s evolution in the phase-space (e.g. during the 3rd January and 19th April 2018 we have discussed above), distinguishing physical dynamics and human control.

A limiting factor for our framework is the long-term background changes that limits the usage of longer-time datasets. For example, changes in the timetable in railways, or governmental policies in economic markets, may significantly impact the dynamics in the phase-space, and would be difficult to filter out of longer-time data. This is also the reason we only use one year of railway data in our study.

Also, it is important to note that it is difficult to distinguish development and causality *only* from the dynamics of two co-varying patterns (the EOFs in Fig 2). Our framework merely describes how their amplitudes evolve in time with respect to each other. It may also be the case that when applying this to other systems, the dynamics cannot be captured well by only two principal components, requiring the usage of higher dimensional phase-space. More local and sequential interactions may be recognized before large-scale disruptions occur. Note, for example, that line L1 and L2 dominate the variance (returning in both EOF1 and EOF2), while in practice, L1 is known to be a relatively disconnected line with a large amount of local (technical) problems. Incorporating more local effects might give more insight in the dynamics of the system. Using only two principal components, however, does trade precision for more significance in our statistical analysis, as the (more) local features may be quite case-dependent.

Another limitation of our framework is that the (sub)clusters found by the Louvain method are only an approximation of sets that the system is likely to remain within, but not well-defined semi-invariant sets (e.g., see [18]). The problem with railway data is that disruptions are relatively short-lived and the probability density function across the phase-space does not show clearly defined states beforehand, but merely focuses on the area around (0,0). This forces us to divide the phase-space into subclusters as we did, while the system can move repeatedly in and out of them. This is visible in Figs 7 and 8, where in both cases the system leaves the subclusters again in 1-2 hours (and not remaining there longer). Moreover, the transition matrix in the first place is used as a Markovian diffusion model on the phase spaces and calculated without taking into account potential memory of the system. Involving memory in the scheme may give more precise clustering and predictions, for example in the case of the depletion of a buffer in rolling stock or crew because of earlier disruption events happening on the same day.

### 5.3 Implications

Although realizing the effects of non-systematic elements and limitations of the framework as described above, it can have strong implications for ST systems in practice.

In general, the framework can be used for any ST system (even for specific non-ST systems where the dynamics are *a priori* not well-known), with the important restriction is that there is enough data of some important dynamic variable. In particular, the framework is useful for heterogeneous spatio-temporal data (e.g., on a network), because it focuses on the dynamics among dominant patterns in space. For example, if one would want to use this to predict the spread of infectious diseases in a particular part of Africa (e.g. related to [47]), one would need enough data of spreadings in the past in that area to find out the most important areas (e.g. cities) that played a role in the spreading, and to build a transition matrix within the phase-space. One could also imagine the evolution of physical systems that are prone to human interaction, for example the evolution certain populations of hunted species, which (if there is enough data) may allow the use of this framework.

Our focus on railway systems brings us to more specific implications for *railway companies*. The framework can be used for real-time monitoring, in which the current position in the phase-space is tracked and the prediction scheme is used to assess the likelihood of entering the disrupted region. If practitioners would use this to anticipate a large-scale disruption, they may act to prevent it. One would start by deriving the dominant principal components. These patterns are an interesting result in themselves, depicting (anti-)co-occurrence of delay in parts of the network as parts with high coefficients (cf. Fig 2). Apparently these patterns are persistent delay configurations and play an important role in the amplification of delay. There may very well be an operational or infrastructural reason behind this.

Specifically, railway traffic controllers can take into account the alarms that are given by the framework (upon construction of the reduced phase-space, followed by the identification of the states). To make the best use of it, an appropriate parameter estimation should be given, which is summarized in answering three questions:

- What time-accuracy should the prediction have? This gives the parameter *ϵ*: how far off the prediction is allowed to be?
- How far ahead of time should it predict, traded for accuracy? (This yields the parameter *t*_max_.)
- What risk does one want to take? In other words, up to what probability does one want to be sure that the system is not (starting to) amplifying towards a disrupted state? (The parameter *p*_*c*_ is 1 minus that probability.)

Upon having chosen these parameters, one can react accordingly to alarms that are given. It is important to stress that the framework’s prediction time horizon is short-term (about 1-2 hours) for the Dutch system. This is both a strength and weakness. The strength is that the framework can be applied real-time, using information of the whole network (rather than delay propagation methods at the micro-scale). The weakness of the short-time horizon is of course related to the limited time left to take intervening measures.

Having discussed the implementation of the framework, the predictions still involve a significant amount of false alarms. There is potential to reduce this by incorporating more operations-related quantities like details on personnel, positions of trains and more local features (rather than purely the macro-sized PCs) into the prediction scheme. Another direction of importance to railway practitioners is to make the prediction more specific in space—information about on which train line or part of the network the delay actually propagates can be valuable information to prevent the disruption’s spread. Note that now, we only do predictions among the first two PCs, which combined only give a rough estimation of where the delay is currently situated. Nevertheless, we believe this paper provides a first-order framework to analyze and predict macro-scale delay evolution in an unconventional manner.

## Supporting information

S1 Appendix Data processing. (PDF)

S2 Appendix Sensitivity analyses. (PDF)

S3 Appendix Case study of a regular day. (PDF)
