# Supplementary material for: Predicting transitions across macroscopic states for railway systems
Source: PLoS One. 2019 Jun 6;14(6):e0217710. doi: 10.1371/journal.pone.0217710 (PMC6553730; doi:10.1371/journal.pone.0217710)
Supplement: S1 Appendix — (PDF) [file pone.0217710.s001.pdf]

S1 Data processing

This section concerns processing of the discrete logging data from the Dutch railways to continuous time series.

S1.1 Data aggregation

We start by defining 'nodes' as the points at which logging takes place, and 'segments' as the tracks between them. In the Dutch railway system, not only the amount of delays are logged, but also whether they concern departures, passings or arrivals. To make the data uniform over the entire network, at any given time, we aggregate all logging activities on to the segments that the trains should be traveling on. Precisely: we aggregate the departure and arrival activities respectively on the segment that the designated train moves to and where it comes from, in the following manner (see Fig. S1.A).

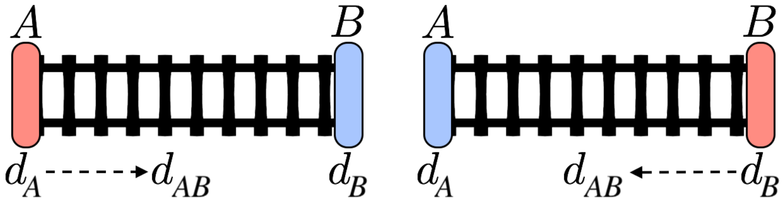

Fig S1.A. Transformation of logged delay at nodes to segmental delay. Consider a train travelling from node A and to node B, where delays d_A and d_B are logged. Left: departure logging at A, meaning that we 'forward' this logged delay in space towards segment AB. Right: arrival logging at B, we aggregate this event also to segment AB.

The choice of dealing with delays on segments (instead of nodes) is motivated in threefold. First and foremost, to improve on spatial precision during aggregation: at larger stations, where many different train lines cross, we would lose information about the direction of the delay propagation if we summed up all delays for the incoming and/or the outgoing trains into one node. Secondly, delays mostly change on the segments themselves. Only in specific cases (e.g., crew transfers, blocking of parts of a station), changes in delay actually take place at a node. (Consequently, the logged activities almost always relate to movements of trains, except for departures at large stations where there is a planned gap between arrival and departure.) The third reason for the choice of segments to aggregate the time series on is that it facilitates easy visualization.

The logs are discrete events. To get a real-time progression of delays on the network, we need to process the logs to generate continuous time series data. At the logging events (e.g., a departure event at node A in Fig. S1.A), both the planned (t_p) and realized (t_r) times are recorded. At the moment of logging, the delay carried by this train is obviously $t_r - t_p$. But between t_r and t_p, the train is already delayed and consequently affecting the segment, although the train is yet to enter the segment. This leaves us with two choices to deal with the delay: (a) keep the delay with the train, i.e., keep it on the segment where the train presently is, or (b) put the delay on the segment where it is planned to be. We choose the latter, since it more accurately indicates the effect of the delay on the network: e.g., when a train is an hour late at a given segment, it already causes problems before it actually gets there (an hour later than scheduled).

Mathematically, we define delay as explained in Eqn. 6 and 7 in the main text. In this form, the equation for $d_i(t)$ contains a discontinuity when t reaches t_r. This is unwanted, since for prediction purposes, we need to treat the system as a continuous dynamical system. We therefore introduce a Gaussian weighted running window with

two parameters: the Gaussian variance σ and a parameter tuning the width of the window p:

$$\bar{d}_i(t) = \frac{\sum_{t'=t-p\sigma}^{t+p\sigma} d_i(t') \cdot e^{-\frac{1}{2}\left(\frac{t'-t}{\sigma}\right)^2}}{2p\sigma + 1} \tag{1}$$

We use $p = 5$ and $\sigma = 3$ seconds, tuned to give a relatively smooth curve without losing too much detailed information. This means that the aggregation is done on a 5-second time resolution, which, for computational reasons, is further reduced to 1-minute time resolution, implying that the precise values of σ and p do not affect the results. In Fig. S1.B we show a smoothed sample time series from 4 February 2018, wherein the distinctive (smoothed) saw-tooth pattern is clearly visible.

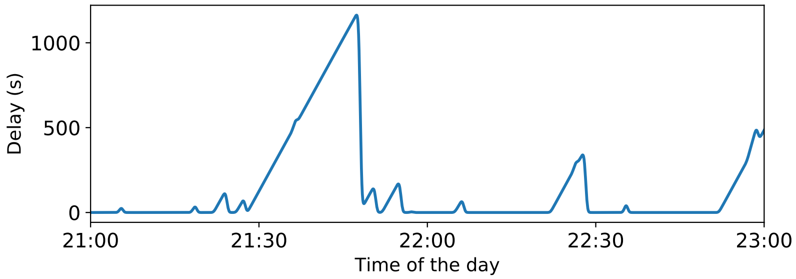

Fig S1.B. Delay for the segment from station Utrecht Vaartsche Rijn towards station Utrecht Central Station, between 21:00 and 23:00. This particular data is from 1 July 2017.

S1.2 Problems with data during large disruptions

In case of very large disruptions, errors in the data logging occurs due to information loss on the exact locations of trains. Another source of errors is the large-scale stand-still of trains, while not all trains are registered as 'canceled'. A consequence of these errors is that trains that are restarted after a network-shutdown are sometimes treated as if they are merely delayed (rather than being canceled and restarted). This results in unrealistically high delay values (sometimes over 13 hours). An instance of this situation occurred on January 18th 2018, also considered as one of the case studies in [21]. On this particular day, trains stood still for a full afternoon without being cancelled, and were simply restarted.

Even for a dataset consisting of 365 days, these strong but erroneous delay sources have a major impact on the PCA due to their large impact on the variance, while they clearly do not play any dynamical role whasoever. In order to remove these spurious events, each train activity that is registered to move with a delay larger than three hours are considered first canceled, and then replanned as a 'new' train such that it loses its delay signature.

S1.3 Classification of days

To get a rough indication of the performance of the system, the manager of the main railway network in the Netherlands (ProRail) uses a day-to-day labeling of each day. The labeling is based on two variables: punctuality and cancellations. These metrics are calculated on a fraction of all train activities. First, the focus is only on the arrival activities at the final destinations, making sure that each train activity is counted only once. Secondly, only passenger (and no freight) trains are considered. Thirdly, a specific

subset of service control points (SCPs) are evaluated, but not all of them. This subset is chosen by expert judgment and in communication with the Dutch ministry of Infrastructure, based on impact on the infrastructure, personnel stress level and passenger flow, and is based on the coverage of lines such that only important stations (in terms of where delay can easily arise, spread or change) are considered. Again, this is motivated to not count a delayed train passing through many SCPs more times than trains that are travelling through less SCPs. Finally, The delay data at the specified service control points is only calculated a few times per day per train line.

Table S1.A shows the specified values per label. Formally, the black days are a subset of red days, but we separate these for analysis purposes.

The SCPs considered are the following (Dutch) passenger stations: Rode School, Groningen, Leeuwarden, Delfzijl, Bad Nieuweschans, Winschoten, Veendam, Harlingen Haven, Sneek, Stavoren, Emmen, Coevorden, Hardenberg, Almelo, Oldenzaal, Hengelo, Enschede, Zwolle, Raalte, Kampen, Deventer, Apeldoorn, Zutphen, Winterswijk, Doetinchem, Nijmegen, Arnhem, Venray, Venlo, Roermond, Sittard, Heerlen, Maastricht, Maastricht Randwyck, Kerkrade Centrum, Eindhoven, Tilburg, 's-Hertogenbosch, Geldermalsen, Tiel, Ede, Barneveld Zuid, Amersfoort, Almere, Utrecht, Gorinchem, Dordrecht, Roosendaal, Vlissingen, Rotterdam, Gouda, Amsterdam Zuid, Amsterdam, Hoorn, Alkmaar, Haarlem, Schiphol Airport, Alphen aan den Rijn, Leiden, Den Haag Centraal and Den Haag HS.

Table S1.A. Day-to-day severity labels by Dutch asset manager ProRail. The last column shows the amount of days in the dataset used in this study (July 1st 2017 - June 30th 2018). Note that the label 'Neutral' applies when none of the other labels does.

Label	Punctuality	Cancellations	Amount of days
Green	>92.5%	<1%	46
Neutral	-	-	292
Red	<85%	>5%	21
Black	<75%	>10%	6

S1.4 Choosing the relevant principal components

Figure S1.C shows the distribution of the explained variance and autocorrelation decay time scale for the first ten PCs, explaining our choice for using only the first two components for the reduced phase-space. As mentioned in Sec. 3, we perform the PCA on the data from the 'disrupted days', i.e., the red and the black days combined together.

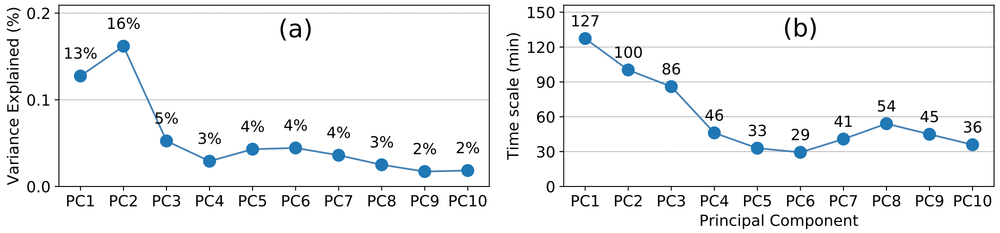

Fig S1.C. Variance explained (a) and time scale of autocorrelation decay rate (b) for the first ten principal components (PCs).

The explained variance (upper panel) by the first two PCs is distinctively more than for the other components, while the persistence (lower panel, defined as the time scale

for the decay of the autocorrelation function) of the first three PCs stands out. Because the third PC contains so little variance, we retain only on the first two PCs to construct the reduced phase-space.
