# Supplementary material for: Predicting transitions across macroscopic states for railway systems
Source: PLoS One. 2019 Jun 6;14(6):e0217710. doi: 10.1371/journal.pone.0217710 (PMC6553730; doi:10.1371/journal.pone.0217710)
Supplement: S2 Appendix — (PDF) [file pone.0217710.s002.pdf]

S2 Sensitivity analyses

The choices we have made for p_c, ϵ and $t_{\max}$ in this paper are 0.08, 30 mins and 90 mins, respectively. The choices should not be based on optimizing the PSS alone, as they are system-dependent: some systems require more time accuracy in practise (thus forcing ϵ to be small), while other require a strong reduction in false alarms (lowering p_c). This sections is devoted to analyzing the results for various choices made in this paper (among which the values of the mentioned parameters). Summarized, it follows that the results are robust and that an intermediate value of the parameters often suffices.

S2.1 Principal components

Since the PCs — calculated from the data on the 'red' and the 'black' days — form the backbone of our analysis, their choice must be robust. In order to ensure this within the scope of the full dataset, with respect to various time resolutions, periods and delay severity labels (Table A in S1 Appendix), we compare the results of the first 10 PCs to those calculated from the full dataset (365 days, 1-min time resolution) as a reference, using Pearson's correlation coefficient. Note that in this comparison between the two sets of PCs, their order (decreasing in variance within each set) may not be the same; hence, the correlations shown are obtained without maintaining the order of the PCs, but maximizing these correlations within the first 25 reference PCs. The results are shown in Fig. S2.A.

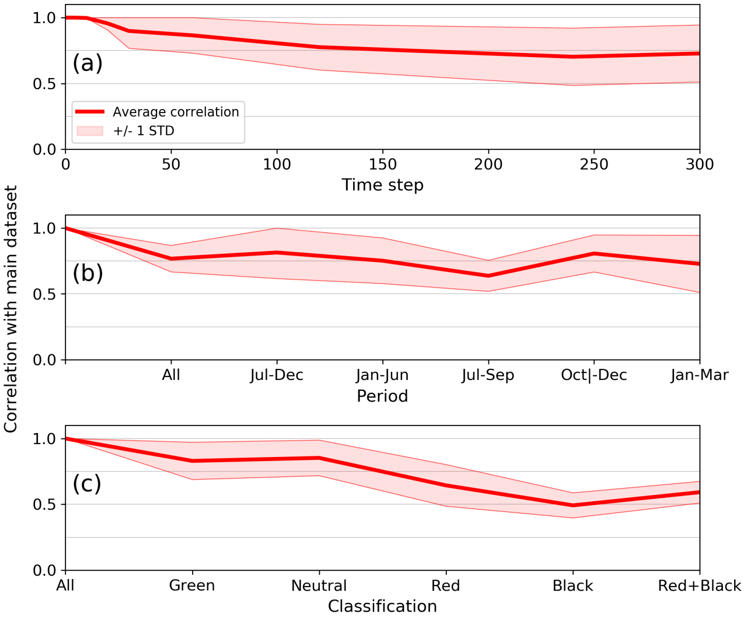

Fig S2.A. (Absolute) correlation of the coefficients of the first 10 PCs with the main dataset over various (a) time steps, (b) periods and (c) day-classifications. Average is shown in thick red line, shaded areas reflect +/- one standard deviation. The order of the eigenvectors is optimized in terms of correlation with the main dataset.

Figure S2.Aa concerns robustness of the PCs with respect to time resolution. For time steps between 1 and 60 minutes, the correlation with the main dataset remains above 0.8 on average, presenting strong evidence of time step robustness. The high robustness up to 60 minutes can be explained by the fact that large amplification (implying most variance) happens on timescales of about an hour. Decreasing the time

resolution also increases the amount of variance explained by the first four PCs (not shown), as a result of the fact that long-(time-)scale variance is more structural than short-scale fluctuations.

A second PC robustness check is performed with respect to period within the one year of data; the results of 2 half-year sets and 4 three-months sets are compared to the whole year, providing insight in non-stationarities related to seasonal variation and changes in the time table. The resulting correlations are shown in Fig. S2.Ab. The average correlations remain above 0.7, reflecting sufficient robustness. The period October to December seems to correlate the weakest, possibly related to the weather.

The third robustness check focuses on the severity labels (see Table A in S1 Appendix), with results shown in Fig. S2.Ac. The PCs of the green and neutral sets compare best with the main dataset, which makes sense as these are the largest sets. However, this might also point to a bias (of the main set) to describe common small fluctuations rather than large disruptions. Overall, the green, neutral and red sets perform reasonably well, with averages of 0.83, 0.85 and 0.64, respectively. The black days correlate worst, with an average of 0.49, indicating that these black days (few as they are) are only weakly represented by the current principal components.

S2.2 Clustering

The (sub)clusters calculated in Sec. 3.2 need to be checked for robustness against gridding (resolution, grid size and symmetric-logarithmic gridding) and the time lag chosen for the transition matrix that formed the basis for clustering.

The results of the clustering with respect to various choices of the grid resolution is shown in Fig. S2.B. The similarities across different resolutions are self-evident, as long as one does not choose too low or too high resolutions. We therefore decided to stick to the middle range, i.e., 123×123.

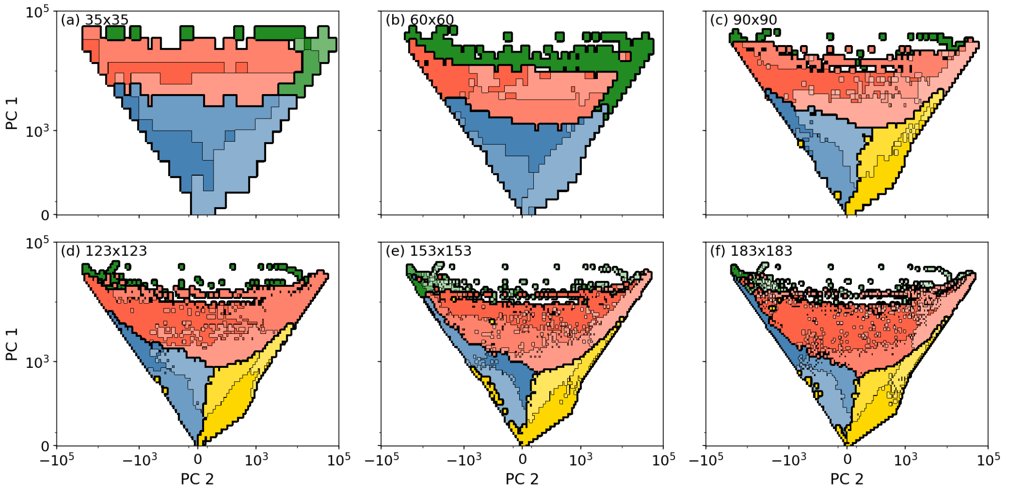

Fig S2.B. Phase-space, containing clusters in colors, and subclusters in various shades of the same (arbitrary) color using transition matrices of various grid resolutions. Areas where there are realizations have been colored. The used value of time lag τ is 30 mins.

Figure S2.C shows the clustering for various time lags used in calculating the transition matrix. Here too the similarities across different time lags are self-evident, as long as one does not choose too small or too large time lags. We therefore decided to stick to the middle range, i.e., 30 mins.

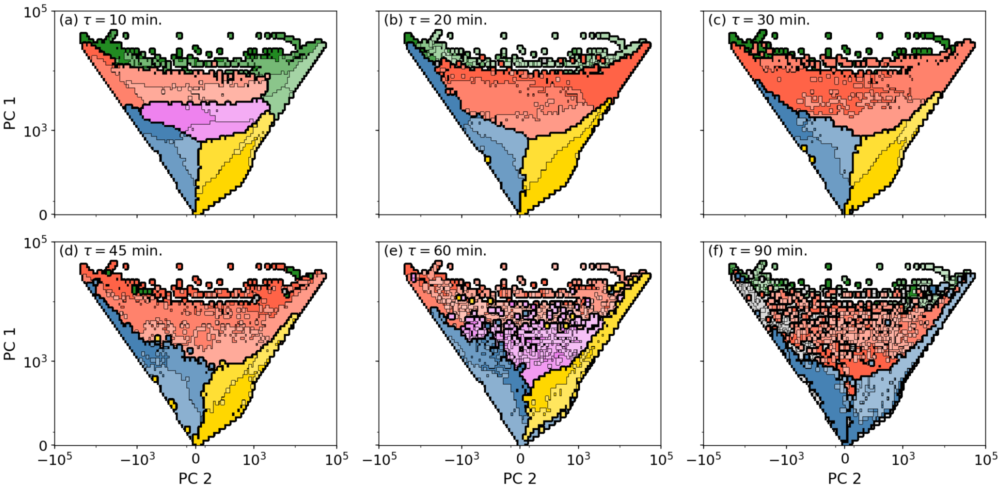

Fig S2.C. Phase-space, containing clusters in colors, and subclusters in various shades of the same (arbitrary) color using transition matrices of various time lags. Areas where there are realizations have been colored. Used grid resolution is 123x123.

S2.3 Alarm lags

We continue with checking how various choices of the critical probability affect the time lag τ_{alarm} at which an alarm is given, and in turn, whether an alarm is given at all within the time horizon $t_{\max}$.

Figure S2.D shows the τ_{alarm} for $p_c = 0.01$ and $p_c = 0.25$, to compare with Fig. 6 of the main article (where $p_c = 0.08$). For $p_c = 0.01$, almost the whole phase-space is colored, indicating that, with high enough $t_{\max}$, alarms are practically always given, which is not useful. When p_c increases to 0.25, we see that only a very small part of the phase-space is colored, limiting predictions only to short times. Intuitively, all of this makes sense: long-term predictions are simply more uncertain. We therefore need to choose p_c somewhere in the intermediate range.

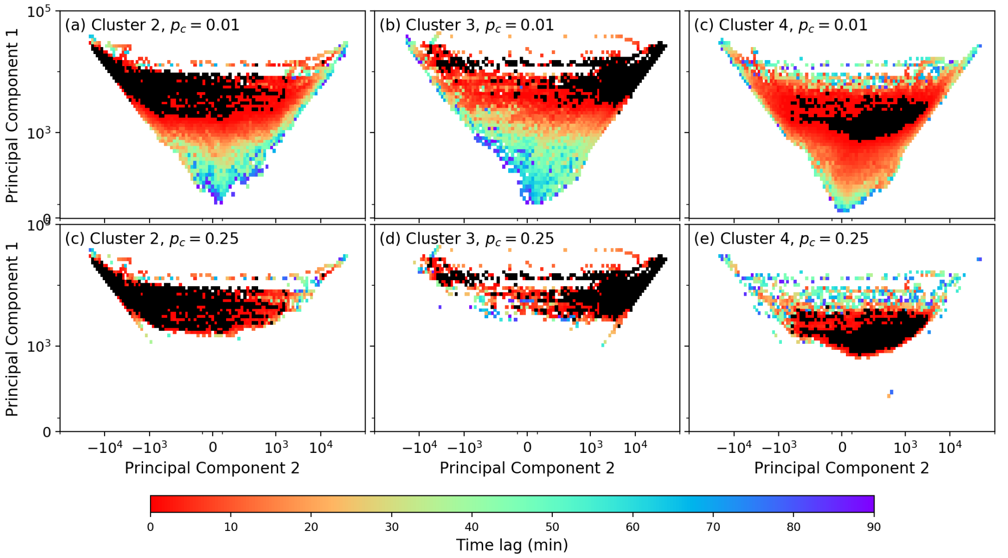

Fig S2.D. Same plots as in Fig. 6 in the main article, but for (a-c) $p_c = 0.01$ and (d-f) $p_c = 0.25$.

S2.4 Parameter sensitivity of Peirce skill score

The parameters ϵ, p_c and $t_{\max}$ have a great modulating impact on the skill score.

Figure S2.Ea-c show the dependence of the skill score on ϵ and p_c. The lower ϵ is, the more precise the predictions are in time, but at the cost of skill (i.e., less alarms when there should be alarms). The score also decreases for higher values of p_c (this can be understood as: there will be a lot more missed alarms, since only a few grid cells will give an alarm at high p_c; see Appendix B.3). In other words, there is an optimum for the skill with respect to p_c.

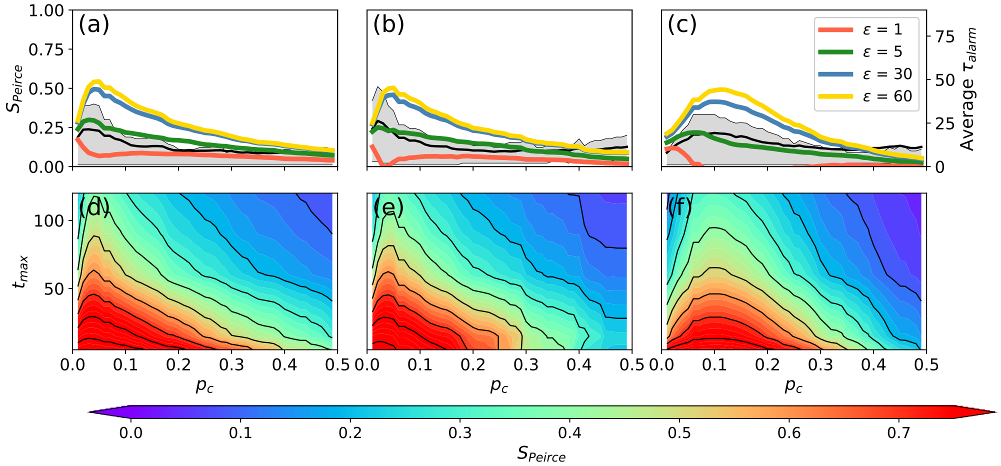

Fig S2.E. Peirce Skill Score for the predictions done towards entering subclusters (a,d) 2, (b,e) 3 and (c,f) 4. Top: Peirce Skill Score for different values of p_c (x-axis) and ϵ (in colored solid lines, legend). Black line (right axis) indicates the median lag at which an alarm is given (i.e., average τ_{alarm}, right axis), including a band width of 25% and 75% percentiles, shown in gray shading. A time horizon of $t_{\max} = 90$ minutes is chosen. Bottom: Peirce Skill Score for different values of p_c and $t_{\max}$. A band width of $\epsilon = 30$ minutes is chosen.

Figure S2.Ed-f reveal the dependence of the skill on the time horizon $t_{\max}$ and p_c. A small time horizon means that one only needs to predict instances of disruption shortly beforehand, which is intuitively a more easily achieved. This is visible in the much higher skill scores at low values of $t_{\max}$. Again, the feature of increasing, then decreasing skill with respect to p_c is visible in these figures.

Another feature visible in Fig. S2.E is that the skills score curves of subclusters 2 and 3 do not differ much with respect to each other, while the curves of subcluster 4 are different: primarily in the form of the shift of the optimum toward higher p_c values. This can explained by the fact that false alarms are important in the signal of this subcluster: for a p_c value of 0.08, alarm is given already at 79% of the phase-space. An increase in the value of p_c reduces the part of the phase-space where an alarm is given, which also reduces the false alarms.

A final feature in Fig. S2.Ea-c is the median τ_{alarm}. Up to $p_c \approx 0.15$, more than half of all alarms given are above 15 min. This is connected to the choice of ϵ, which should be smaller than the alarm lag given. If this is not the case, then the alarm's precision in time becomes arbitrary.
