# Supplementary material for: Predicting transitions across macroscopic states for railway systems
Source: PLoS One. 2019 Jun 6;14(6):e0217710. doi: 10.1371/journal.pone.0217710 (PMC6553730; doi:10.1371/journal.pone.0217710)
Supplement: S3 Appendix — (PDF) [file pone.0217710.s003.pdf]

S3 Case study of a regular day

Here, we discuss the performance of the prediction scheme if there are no instances when the system reaches the disrupted state. Figure S3.A shows the system's trajectory in the phase-space on 27th April 2018, along with all prediction outcomes towards subclusters 2 and 3.

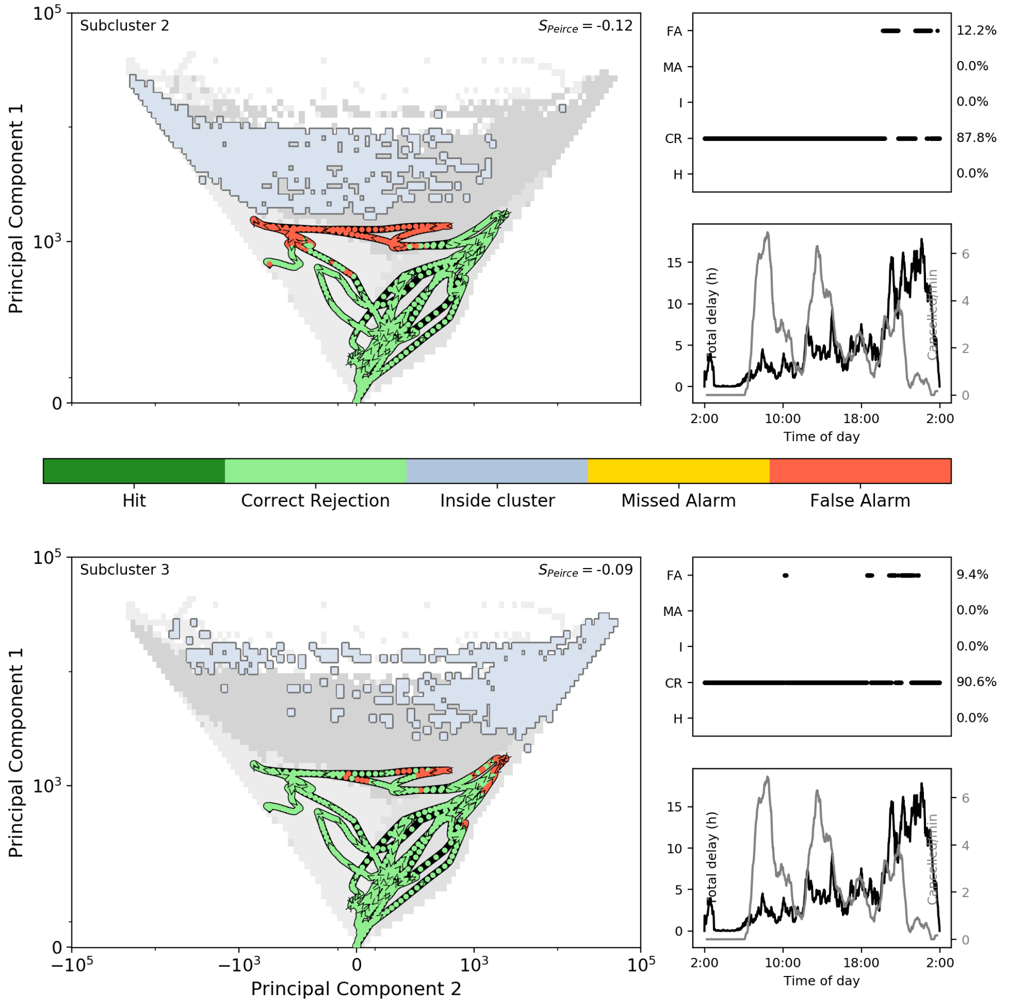

Fig S3.A. Same as in Fig. 7 of the main article, but for the case of 27th April 2018.

The system does not attain high PC1 values and remains within the lower subclusters, mainly in the positive PC2 area (i.e., delays mostly on L1 and L3). It does not reach subclusters 2 and 3 at all, but does enter subcluster 4 twice (as noted earlier, this is common for regular days). In the right panels, we see that in the night or early morning, the system stays 'quiet': correctly, almost no alarms are given, resulting in a lot of correct rejections (blue dots). Later in the day, delay builds up and after a lot of false alarms, the metric correct predicts ('hits') the entrance to subcluster 4.

Correct predictions to enter subcluster 4 are made up to 56 minutes in advance, with a band width of $\epsilon = 30$ minutes. The time horizon $\tau_{\max}$ is 90 minutes, so there are no correct predictions between 56 and 90 minutes prior to the entrance of the subcluster. This actually reflects the situation on regular days: the system may touch upon, or even reach, subcluster 4, but it remains difficult to predict. As the choice of $p_c = 0.05$ is

relatively low, a lot of false alarms (58%) are given, while no missed alarms are registered. The absence of missed alarms combined with the occurrences of hits maximizes the ratio #H/#O (i.e., it becomes 1), increasing the Peirce skill to a positive score despite the number of false alarms. However, while predicting the entrance in subclusters 2 and 3, we obtain a negative PSS due to the fact that no Hits are predicted (it never reaches these subclusters).

The right panels of Fig. S3.A show the evolution of the total delay and cancellations. For cancellations we use the amount of train activities (departure, arrival, short stops etc.) that were scheduled, but canceled, per minute. This is a measure of the reduction of 'stress' on the system by human decision. Note that there is a strong increase in total delay roughly from 19:00 onwards, coinciding with the entrance to subcluster 4. The signal in cancellations per minute is however difficult to interpret. It seems that during two events, roughly at 06:00 and 13:00, there were a lot of cancellations; we could not retrieve the reasons behind these cancellations. It could be that these cancellations avoided the entrance to subcluster 4, by limited the system's overall delay.
